# Genome editing in grain legumes for food security

**DOI:** 10.3389/fgeed.2025.1572292

**Published:** 2025-05-20

**Authors:** Joshua Yeboah Asiamah, Sakina Haruna Mahdi, Kusum R. Tamang, Christian Bryan Carson, Prabesh Koirala, Emily Anne Reed, Aaron Tettey Asare, Anu Augustine, Milind B. Ratnaparkhe, Kailash C. Bansal, Babu Valliyodan

**Affiliations:** ^1^ Department of Agriculture and Environmental Sciences, Lincoln University, Jefferson City, MO, United States; ^2^ Department of Molecular Biology and Biotechnology, University of Cape Coast, Cape Coast, Ghana; ^3^ Department of Biotechnology and Microbiology, Kannur University, Kannur, India; ^4^ Department of Biotechnology, ICAR-Indian Institute of Soybean Research, Indore, India; ^5^ National Academy of Agricultural Sciences, New Delhi, India

**Keywords:** TALEN, ZFN, CRISPR/Cas9, prime editing, grain legumes, gene-editing, visualization of similarities viewer (VOSviewer)

## Abstract

Throughout history, leguminous crops have contributed significantly to the human diet. Grain legumes have long been identified as a valuable nutritional source for humans. However, their significance extends beyond nutrition to global food security, reducing reliance on chemical fertilizers, improving soil health and increasing resilience to climate change. Recognizing their vital importance in nutrition and agricultural production, scientists have worked persistently to uncover new genetic traits in legumes, resulting in enhanced yields, improved nutritional value and increased stress tolerance. Recently, the availability of genomic resources for new traits in grain legume plants has greatly increased, laying the groundwork for the adoption of advanced breeding technologies. Gene editing has shown significant potential to improve crop outcomes. This review critically examines the latest developments in gene-editing techniques specific to major grain legumes, focusing on their application in enhancing legume crops with significant agronomic characteristics. The article also shows the potential advantages associated with these advancements. Over the years, advancements in technologies such as Transcription Activator-Like Effector Nucleases (TALENs), Zinc Finger Nucleases (ZFNs), Clustered Regularly Interspaced Short Palindromic Repeats (CRISPR/Cas9), and the more recent Prime Editing technique have significantly contributed to genetic enhancements. These innovations have improved nutritional and market traits, boosted farming incomes, and increased the accessibility of affordable nutritious food, particularly in developing nations. Studies show that CRISPR/Cas9 is the most extensively applied gene editing technology in grain legumes. The advent of this technology has transformed genetic modification by offering exceptional precision and efficiency. This progress has enabled the creation of grain legumes that are more resistant to climate change and enhanced with improved nutritional content. Our research highlights that soybeans have been the primary focus of CRISPR/Cas9 gene editing efforts, surpassing any other grain legume, unlocking significant potential for innovation and improvement. This article presents a scientometric analysis of bibliographic data from the Web of Science using VOSviewer. It highlights global research trends, emphasizing China’s leading role in international collaborations, the prominence of soybean (*Glycine max*) in CRISPR/Cas9 studies, and the key researchers driving advancements in gene editing for food security.

## Introduction

As we progress into the 21st century, significant climate change due to human induced effects will lead to environmental degradation which poses a challenge to global food production and ecosystem (Kole et al., 2015; Cai et al., 2020a). It is projected that approximately 840 million individuals worldwide will experience undernourishment by the year 2030 (Mohajan, 2022). Necessary transformations must be made in our food production, processing, and consumption methods to remedy these problems (Food and Agriculture Organization, 2018). Furthermore, a global transition towards vegetarian diets has been recognized as a crucial imperative in addressing issues related to malnutrition and sustainability (Food and Agriculture Organization, 2018; Willett et al., 2019). Recently, there has been advancing exploration for substitutes to animal-based food, leading to increased attention on legumes as favorable choices, due to their numerous favorable economic, social and environmental characteristics (Stagnari et al., 2017). In this time of climate change challenges, breeders have made remarkable progress in developing grain legume varieties that are notably resilient. Yet, the journey towards perfection continues. Experts are now exploring the exciting possibility of breeding innovative climate-smart soybean varieties to use the atmospheric drying phenomenon, enhancing their nitrogen fixation capabilities, and potentially increasing yields and resilience. This promising direction not only highlights the adaptability and ingenuity in agricultural practices but also opens new avenues for sustainable agriculture. As we consider the future and the challenges it holds, the potential of gene editing technologies comes into question. Gene editing (GE) or genome editing (GE) has gradually become an integral part of plant breeders’ tools, garnering worldwide attention for its significant potential in advancing crop improvement initiatives. Techniques comprising TALENs, ZFNs, the CRISPR/Cas 9 system and prime editing are being effectively used in gene editing, offering new prospects for addressing the agricultural challenges of tomorrow. This review offers an updated perspective on the advancements in gene-editing methods for enhancing grain legumes. It discusses the essential requirements and challenges that remain in applying this technology to agriculture. Furthermore, it highlights the contribution of gene editing to legume research and development.

## Importance of legumes

### The “poor man’s meat” as a global food and health security

Legumes rank highly as crucial crops worldwide. Throughout history, grain legumes have been an integral part of traditional diets across diverse cultures. They offer a wealth of nutritional benefits, including protein, starch, dietary fiber, phytochemicals, and micronutrients, while maintaining a low-fat content (Siddhuraju and Becker, 2003). A substantial collection of biochemical, epidemiological, and clinical studies strongly suggests a positive connection between grain legume consumption and a lower occurrence of illnesses, such as cardiovascular diseases, cancer, diabetes, and obesity. (Siddhuraju and Becker, 2003; Vadivel and Pugalenthi, 2008). Studies have also highlighted the potential nutritional value of lesser-known or wild legume seeds and other pulses that hold significance for tribal communities (Vadivel and Pugalenthi, 2008). Recognized for their high levels of protein, fiber, and essential micronutrients, underutilized legumes like *Mucuna pruriens* and *Bauhinia purpurea* have demonstrated significant health benefits and exhibit strong adaptability to challenging environments, particularly due to their drought resistance. Incorporating these legumes into diets can improve food security and enhance nutrition in rural and local communities (Janardhanan et al., 2003; Vadivel and Pugalenthi, 2008). In addition, inadequate intake of essential micronutrients and malnutrition resulting from a lack of dietary protein can lead to conditions such as kwashiorkor, marasmus, anemia, compromised immune function, and environmental enteric dysfunction. These conditions are most prevalent in developing nations and countries with minimal financial resources, such as South Asia and sub-Saharan Africa, earning it the well-known nickname as the “poor man’s meat” (Pinstrup–Andersen et al., 1993; Brabin and Coulter, 2009; World Health Organization, 2004; Food and Agriculture Organization, 2004). A larger share of individuals living in these areas primarily rely on maize, sorghum, rice and cassava as staple foods in their daily diets. While these foods are high in starch content, they lack an adequate protein supply (Levin et al., 1993). Consequently, a significant proportion of the population in these regions, particularly infants, do not meet their daily protein requirements, which can have adverse effects on their growth and development (Asare et al., 2010; Larweh et al., 2019).

Grain legumes are an excellent source of plant-based protein and have various medicinal properties. They contain proteins that help regulate sugar and water levels, and metabolism, while supporting reproduction and body and brain development. Research has shown that phytosterols are found in grain legumes (soy, peas, and beans) (Ryan et al., 2007; Singh et al., 2021). Phytosterols have antioxidant properties and compete with fats in the gut for absorption due to their phytochemical composition resembling cholesterol (Ryan et al., 2007; Singh et al., 2021). Phytosterols are recommended for conditions including breast cancer and osteoporosis (bone loss), as well as for lowering blood cholesterol levels (Messina, 1999; Salgado and Donado-Pestana, 2011; Singh et al., 2021). Legumes also possess diuretic effects, although adding fat and salt can diminish this benefit (De and De, 2019; Yao et al., 2020). Egyptian studies have shown that foul beans (fava beans) have beneficial effects in reducing diarrhea in infants, thereby lowering infant mortality rates (Wells, 2016). Furthermore, these beans have been found to help regulate blood sugar levels in individuals with diabetes. Chickpeas, known for their positive effects on the pancreas and stomach, have a higher iron content compared to any other grain legumes. They also contain unsaturated fats (Wood and Grusak, 2007). In China and Japan, soybeans are used to clear fat deposits from the blood, improve pancreatic function, and detoxify the intestines (Jayachandran and Xu, 2019). Soybeans and soybean products have various advantages, including increasing milk production during nursing, reducing hypertension during pregnancy, addressing malnutrition, and providing brain nourishment through lecithin (Zaheer and Humayoun Akhtar, 2017; De and De, 2019).

Beans are considered a staple in the American diet, in most Latin American diets, and generally worldwide (Berglund-Brücher and Brücher, 1976; Nabhan, 2016). Due to its affordability, beans are often unfairly labelled as a ‘poor man’s meat’, especially in sub-Saharan Africa (Asare et al., 2010; Samaranayaka, 2017; Asiamah, 2020). It may no longer be regarded as “poor man’s meat,” but rather as a nutritious and healthy alternative to conventional meat. Thus, grain legumes are a helpful fragment of the world’s food and health security. It is said ‘if you are looking to cut back on red meat, consider adding more beans to your diet’ (Schofield and Henderson, 2015).

### Grain legumes: nature’s nitrogen-fixing powerhouse

The Romans recognized the benefits of legumes in animal feed and soil improvement as early as 37 B.C., appreciating their nitrogen-fixing abilities (Vasconcelos and Gomes, 2016). Nitrogen is a major element necessary for the development of plant biological structures. Plants require an adequate supply of nitrogen for optimum growth and development, which directly influences crop yield and quality. Adequate nitrogen availability is vital as it affects not only the yield but also the nutritional composition of plant-based food products, which serve as feed for both animals and humans (Peoples et al., 2021). Plants have two primary ways of acquiring nitrogen: through root assimilation or, as in legumes, through atmospheric fixation (Sulieman and Tran, 2015; Kiba and Krapp, 2016). Nevertheless, farmers rely heavily on synthetic nitrogen fertilizers derived from fossil fuels to improve agricultural production. This dependency on chemical fertilizers is driven by the goal of maximizing crop yields and ensuring profitable harvests. With the global population projected to exceed nine billion by 2050, there is an urgent need to increase annual legume production by at least 70% over the next 3 decades (Dave et al., 2024). This growth is essential to address the escalating global demand for food security (Yitbarek, 2019). Hence, a critical question arises: How can we effectively manage or minimize the application of synthetic nitrogen fertilizers while advancing sustainability in the context of the Next Green Revolution or food security? Grain legumes are known for their remarkable ability to convert atmospheric nitrogen gas (N_2_) into organic nitrogen, essentially making their own fertilizer out of thin air, providing vital nourishment for the next crop. Legumes, in comparison to industrial nitrogen fixation, offer valuable ecosystem services and function as environmental “guardians.” They contribute by decreasing reliance on synthetic nitrogen fertilization, promoting soil conservation, and fostering diverse and biodiverse agricultural systems. With the expected transformations in the American and global food sector, it becomes clear that dedicated efforts should be directed towards the production and improvement of grain legumes. Over the last 20 years, there has been a significant rise in applied and basic scientific research focusing on various model legumes, including soybeans (*Glycine max*), a grain legume whose genome was recently fully sequenced (Stupar and Specht, 2013). These investigations have yielded valuable findings, particularly in reference to symbiotic nitrogen fixation-associated genes present in soybeans. Notable genes include signaling receptors such as *GmNFR1β* and *GmNFR1α*, which function as LysM receptor kinases and NF receptors (Stracke et al., 2002; Endre et al., 2002; Madsen et al., 2003; Arrighi et al., 2006; Smit et al., 2007; Indrasumunar et al., 2010; Indrasumunar et al., 2011; Indrasumunar et al., 2015). Additionally, genes associated with nodule signaling and nodule organogenesis, for instance *GmSYMRKα* and *GmSYMRKβ*, function as leucine-rich repeat receptor kinases (LRR-RK) that are fundamental for both root nodule and mycorrhizae symbiosis (Sulieman and Tran, 2015; Kiba and Krapp, 2016; Peoples et al., 2021). Moreover, autoregulation of nodulation (AON) genes like *GmNARK* and *GmRIC1/2,* functioning as LRR receptor kinases, play a pivotal role in the systematic regulation of nodule numbers. Finally, small peptides derived from roots have been identified as long-distance signaling (Nishimura et al., 2002; Krusell et al., 2002; Searle et al., 2003; Schnabel et al., 2005; Gresshoff et al., 2025). Root-derived small peptides are crucial for long-distance signaling in plants, especially for adapting to nitrogen (N) availability fluctuations. This research has demonstrated that during nitrogen starvation, rootlets secrete small peptides that travel to the shoot, where they are detected by two leucine-rich repeat receptor kinases (LRR-RKs). This signaling pathway is vital for the plant to adjust its root development and nitrate uptake in response to localized N deficits. Arabidopsis plants lacking this signaling mechanism show growth retardation and symptoms of N deficiency. CRISPR-Cas9 has been successfully utilized to enhance nodulation in grain legumes, optimizing nitrogen fixation for improved soil health and sustainable crop production (Table 4).

## Gene editing technologies as a sustainable approach for enhancing food security

The study of gene editing *in vitro* has been ongoing since the 1970s when it was found that exogenous DNA could be taken up by bacteria or yeast and incorporated into the genome of interest. This was later followed by a demonstration of integration of DNA into the genome of the yeast *Saccharomyces cerevisiae* (Scherer and Davis, 1979). In this process, a specific gene of interest is precisely targeted to alter its function. A nuclease, an enzyme that cuts nucleic acids, is employed to cleave the DNA sequence of that gene, thereby disrupting its structure. Following the cleavage, a new gene can be inserted, alterations can be made to existing sequence, or specific segment of genomic DNA can be deleted. The field of genome engineering has advanced with the development of highly precise gene-editing tools, including Zinc Finger Nucleases (ZFNs), RNA-Guided Engineered Nucleases (RGENs), and Transcription Activator-Like Effector Nucleases (TALENs) (Arrighi et al., 2006). However, the groundbreaking introduction of CRISPR-Cas system technology has garnered global attention. This innovative editing system, along with other gene editing tools, operates based on three fundamental biological mechanisms, as noted by Sauer et al. (2016) and Songstad et al. (2017). These mechanisms are: first, the ability to precisely identify specific DNA sequences; second, the capability to cleave DNA at those exact locations; and third, the engagement of the cell’s innate DNA repair machinery. All gene editing tools utilize nucleases, the enzymes responsible for DNA cleavage. In addition to nuclease-based technologies, oligo-directed mutagenesis (ODM) provides another approach, enabling targeted alteration of a specific DNA nucleotides at a desired location (Sauer et al., 2016; Songstad et al., 2017). These tools provide a sustainable approach to improving food security in grain legumes. These tools enable precise modifications to the genetic makeup of grain legumes, potentially leading to improved crop yields, enhanced nutritional value, and greater resilience to environmental stresses. By targeting genes associated with traits like nutrient production, yield potential and stress responses, gene editing can improve resource utilization and promote stable crop yields. Gene editing provides opportunities to address limitations in the nutritional composition of grain legumes, making them more nutrient-rich and accessible as food sources. Moreover, gene editing offers the potential to develop legume varieties better suited to evolving climatic conditions, thereby ensuring consistent and dependable food production.

Currently, there are only a limited number of applications of genome editing techniques, particularly CRISPR-based systems—in both the market and pre-market stages (Metje-Sprink and Mishra, 2020; Parisi and Rodríguez-Cerezo, 2021). This scarcity can be attributed to the relatively recent development of genome editing technologies, coupled with regulatory uncertainties surrounding their use in many countries. Nonetheless, it is anticipated that numerous new applications are likely to arise in the future and eventually become available on the market (Metje-Sprink and Mishra, 2020). As of now, there are a limited number of commercially available genome-edited crops globally. The two most notable examples are high-oleic soybean in the United States and tomatoes enriched with γ-aminobutyric acid (GABA) in Japan. GABA, a naturally occurring compound, has been shown to effectively lower blood pressure (Nonaka et al., 2017). In 2023, mustard leaf enriched with nutrients using CRISPR/Cas technology, offering higher nutrition compared to lettuce, was launched in the U.S. market by the *Pairwise Company®,* with expectations that numerous other genome-edited crops will be introduced in the coming years (Metje-Sprink and Mishra, 2020). High-oleic soybeans provide enhanced oil stability by having increased levels of oleic acid and reduced levels of linolenic acid (Demorest et al., 2016). However, despite its transformative potential, the application of CRISPR/Cas in agriculture requires stringent regulatory oversight to ensure safety and ethical considerations. As genome-editing technologies evolve, ongoing research and adaptive regulatory frameworks are essential to harness their full potential for sustainable crop development and global food security.

### Transcription activator-like effector nuclease (TALEN) application in grain legumes

**FIGURE 1:**
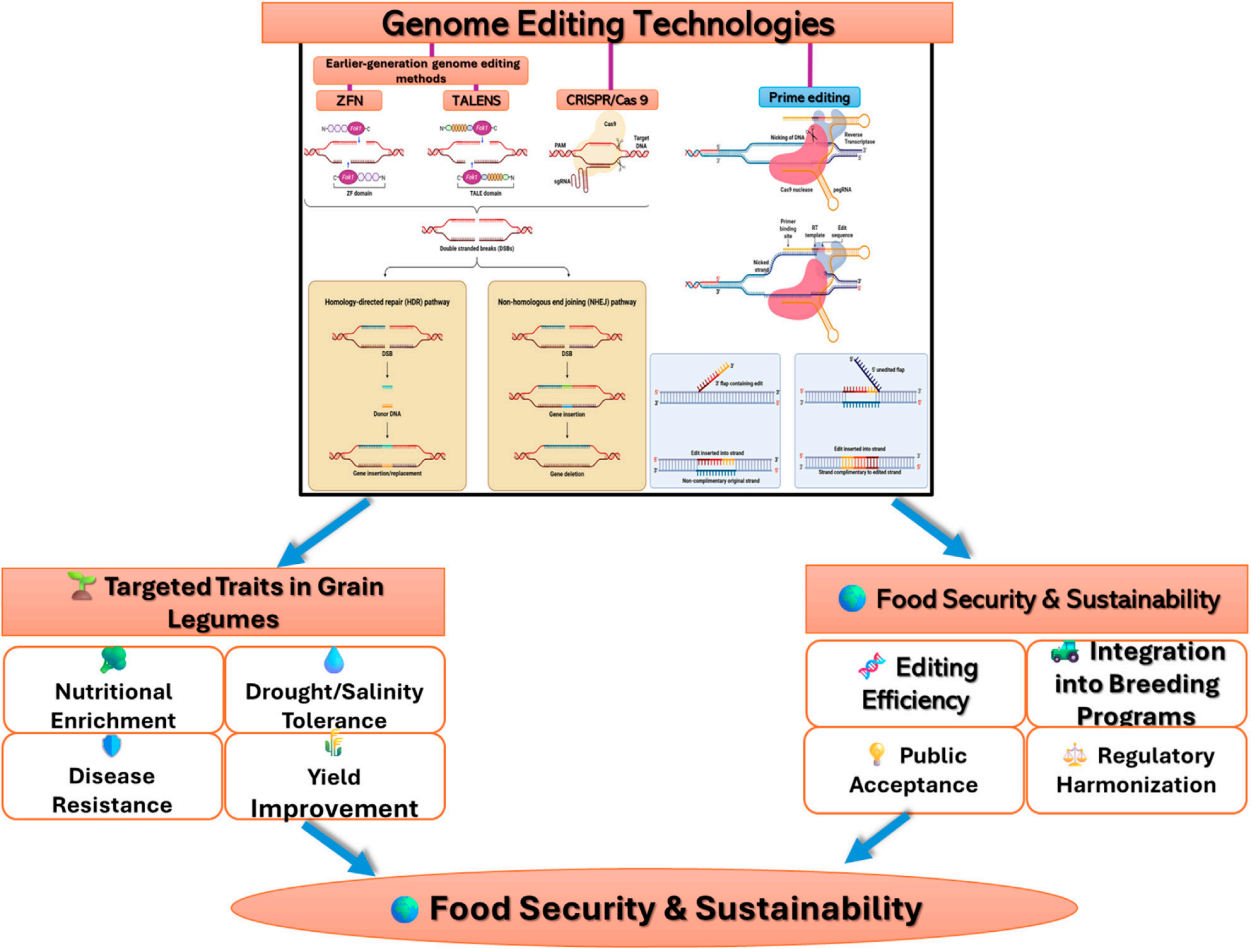
Conceptual overview of gene editing in grain legumes for food security.

TALEN has become a significant instrument in the progress of gene editing, offering remarkable contributions. Originating from the pathogenic bacterium *Xanthomonas*, which is responsible for causing severe diseases in diverse crops, TALEN showcases a fascinating evolutionary background. During the infection process, *Xanthomonas* employs a type III secretion system which introduces transcription activator-like (TAL) proteins within the cytoplasm of the target cells. These TAL proteins function as transcription factors within the host, triggering advantageous developmental alterations in plants that facilitate bacterial colonization during the disease progression. The structural composition of TALs primarily encompasses three essential elements: nuclear localization signals, a central region containing tandem repeats, and a transcriptional activation region (Boch et al., 2009). The tandem repeat regions, typically consisting of 33–35 highly conserved amino acids, serve a pivotal function in DNA binding (Zhang et al., 2014). Target DNA binding specificity is determined by hypervariable residues situated at positions 12 and 13 within the repeat domain. Termed repeat-variable di-residues (RVDs), these residues are associated with the four DNA bases. In the context of TALENs, the activator domain is substituted with *FokI,* thereby transforming TALENs into target-specific gene editing tools (Figure 1). TALENs are used in pairs to generate double-strand breaks via FokI, binding to opposite DNA strands with a spacer region in between (Zhang et al., 2014). Initially, TALENs were designed by linking the *FokI* nuclease to the C-terminal activator domain, but truncating the C-terminal sequences improved TALEN efficiency. However, the application of TALENs in grain legumes for genome editing has shown limited promise, although some attempts have been made in specific legume crops such as soybean (Bedell et al., 2012; Joung and Sander, 2013) (Table 2). TALENs have been successfully used to target the soybean Fatty Acid Desaturase 2 (FAD2) gene to enhance oleic acid production (Table1) (Haun et al., 2014). TAL effectors were successfully employed in soybeans to develop dwarf and albino phenotypes by altering the *GmPDS11* and *GmPDS18* genes (Du et al., 2016).

**TABLE 1:** TALEN-mediated genome editing technology application in grain legumes.

Legume	Delivery method	Gene of interest	Function/trait	Outcome	References
*Glycine max (Soybeans)*	*A. rhizogenes strain K599-* mediated delivery	FAD2	Conversion of oleic acid to linoleic acid changes the fat composition in soybean oil	Higher oleic acid and lower linoleic acid content	Haun et al. (2014)
*Glycine max (Soybeans)*	*A. rhizogenes strain K599*	GmPDS11 and GmPDS18	Involved in the carotenoid biosynthesis pathway	Development of dwarf and albino buds	Du et al. (2016)
*Glycine max (*Soybeans)	Agrobacterium-mediated transformation	Fatty Acid Desaturase genes (FAD2 and FAD3)	Synthesis of fatty acids, influencing oil quality	Improved oil quality, with higher oxidative stability	Demorest et al. (2016)
*Glycine max (*Soybeans)	*A. rhizogenes strain K599*	GmDcl2b	Biogenesis and function of small RNAs	Generated mutations using TALENs; the heritability of mutations was not specified	Curtin et al. (2018)

While TALEN construction is comparatively simpler than that of ZFNs, it lags behind CRISPR technology. Designing a TALEN pair to target a 20 bp gene necessitates the design and assembly of 20 repeat-variable di-residues (RVDs) into a plasmid. These two steps have been described by researchers as a time-consuming and tedious process (Baloglu et al., 2022). Less than this effort, CRISPR technology enabled the construction of a plasmid targeting 10 different genes. However, CRISPR raises ethical concerns and questions. One major issue is the potential for unintended consequences, such as off-target mutations that might disrupt non-target genes, raising safety and environmental concerns. There are also worries about ecological imbalance, especially when gene-edited organisms are released into the wild without thorough long-term studies. In the global South, equity and access emerge as pressing ethical concerns—will smallholder farmers benefit from this technology, or will it deepen existing inequalities in agricultural systems? Moreover, the lack of harmonized international regulations can lead to ethical dilemmas in trade, labeling, and consumer choice (Kuzma, 2018). Lastly, there is public distrust, partly fueled by associations with GMOs, that raises questions about informed consent, transparency, and societal oversight in the use of genome-edited grain legume crops (Ishii and Araki, 2017). Despite this, CRISPR has gained popularity as a genome-editing tool because of its convenience, effectiveness and ability to target multiple genes simultaneously (Jacobs et al., 2015; Song et al., 2024). Nonetheless, there are instances where TALENs are preferred. One key advantage is their ability to target longer DNA sequences, which helps reduce the likelihood of off-target mutations (Haun et al., 2014). In such cases, researchers may opt for TALENs over CRISPR to reduce the minimize the chances of off-target effects. Modern genome editing approaches have significantly advanced in their ability to target longer and more specific DNA sequences while minimizing off-target activity. Tools like TALENs (Transcription Activator-Like Effector Nucleases) offer a distinct advantage by recognizing longer DNA motifs (typically 30–40 bp), which enhances target specificity and reduces the risk of unintended gene modifications or silencing (Haun et al., 2014; Joung and Sander, 2013). This feature makes TALENs particularly valuable in applications requiring high precision, such as modifying single-copy genes in complex plant genomes. Additionally, the CRISPR/Cas system has been refined through innovations such as high-fidelity Cas9 variants (e.g., SpCas9-HF1, eSpCas9) and paired nickases, which drastically reduce off-target cleavage (Slaymaker et al., 2016; Kleinstiver et al., 2016). Furthermore, base editing and prime editing techniques now allow for single-nucleotide edits without inducing double-stranded breaks, further decreasing the chances of off-target effects and off-site silencing (Komor et al., 2016; Anzalone et al., 2019). These modern advancements are enabling researchers to pursue gene editing with a higher degree of confidence and safety, especially in crop species where unintended mutations could have broad agronomic implications.

### Zinc finger nucleases (ZFN) application in grain legumes

The early 1990s marked considerable progress in genome-editing technology. With a more thorough understanding of DNA repair mechanisms, the first targeted genome-editing strategy, Zinc Finger Nucleases (ZFNs), was devised. This technique integrates a zinc finger protein domain with a nuclease domain to enable precise gene editing. Similar to the TALEN method, ZFNs function as pairs to create double strand breaks in DNA, utilizing the FokI enzyme for cutting. The zinc finger (ZF) domain is made up of as many as six protein subunits, each designed to bind to a specific DNA sequence. These zinc finger proteins (ZFPs) are engineered to interact with Zn^2+^ ions, which stabilize their structure, enabling them to bind effectively to three DNA base pairs (Figure 1). Three primary strategies have been implemented to construct ZFNs: (1) assembling modular ZFN domains that allow researchers to select specific sequences and create a ZFN pair; (2) using context-sensitive selection methods to design novel ZFNs tailored to desired targets; and (3) combining pairs of double ZFN domains through computational optimization and pre-existing libraries to achieve specific locus targeting. These approaches have been successfully applied by researchers, as documented in numerous studies. The application of ZFNs has been demonstrated across different organisms, including human cells, and has achieved limited success in legumes. Beyond Agrobacterium-mediated transformation, direct delivery methods like electroporation and nanoparticle-based systems are being optimized for ZFNs in grain legumes. These approaches aim to improve efficiency, especially in recalcitrant species like peanuts (*Arachis hypogaea*), where traditional methods often yield low transformation rates (Ahmad et al., 2021). However, ZFNs face challenges compared to newer technologies like CRISPR/Cas9. They are more complex to design and engineer, which can complicate their development and application. Additionally, ZFNs may exhibit reduced precision compared to CRISPR/Cas9, leading to unintended genetic changes. Even with these limitations, ZFNs have played a role in advancements in genome editing. Nonetheless, the emergence of CRISPR/Cas9, which offers greater efficiency and user-friendliness, has largely replaced ZFNs as the preferred genome-editing tool among scientists. Despite these challenges, Zinc Finger Nucleases (ZFNs) have demonstrated utility in legumes, particularly in soybeans (*Glycine max*), where they have been applied to precisely modify genes like GmLox1 and GmLox2. These edits have been effective in reducing the activity of lipoxygenase enzymes, which is beneficial for enhancing the taste profile of soybean products (Table 2). Additionally, ZFNs have been utilized to target the DCL4 gene, resulting in improved lateral root formation.

**TABLE 2:** Zinc finger nucleases (ZFN)-mediated genome editing technology application in grain legumes.

Legume	Technique	Delivery method	Gene of interest	Function/trait	Outcome	References
*Glycine max* (Soybeans)	ZFN	Agrobacterium-mediated transformation	GmLox1 and GmLox2	production of lipoxygenase enzymes	2 base pair differences in the genes of interest to reduce lipoxygenase activity	Curtin et al. (2011)
*Glycine max* (Soybeans)	ZFN	Agrobacterium-mediated transformation	DCL4a/b	Dicer-like protein	Enhanced lateral root growth	Menz et al. (2020)

Despite these successes, the broader implementation of ZFNs in other grain legumes—such as peanuts (*A. hypogaea*), chickpeas (*Cicer arietinum),* and cowpeas (*Vigna unguiculata*)—has faced significant challenges. These crops have inherently complex genomes, and their resistance to conventional transformation methods has complicated the effective application of ZFNs. To overcome these limitations, research has increasingly focused on refining the techniques used to deliver ZFNs into plant cells. Although Agrobacterium-mediated transformation is still frequently employed, alternative delivery methods—including electroporation, nanoparticle-based vectors, and protoplast transformation—are under active development.

### Gene-edited grain legumes by CRISPR/Cas system

The CRISPR/Cas system, widely recognized as the leading genome-editing tool, has garnered significant attention in the scientific community since its introduction in 2012(Jinek et al., 2012; Menz et al., 2020; Parisi and Rodríguez-Cerezo, 2021). Unlike conventional genetic modification, genome-edited plants using CRISPR/Cas system may not be classified as GMOs, facilitating their regulatory acceptance worldwide (Molinari et al., 2024). Originally derived from bacterial defense mechanisms, CRISPR/Cas consists of a guide RNA (crRNA) that directs the Cas protein to a specific DNA sequence, enabling targeted modifications. The most widely used systems, CRISPR/Cas9 and CRISPR/Cas12a, allow for precise genome alterations, including gene knockouts and base editing. This natural mechanism-based technology has outperformed other genome editing tools, including meganucleases, ZFN technology and TALENs, and was named the groundbreaking innovation of the year in 2013. CRISPR’s remarkable advantages and versatility have propelled it to the forefront of genome editing, enabling precise modifications and revolutionizing the field of genetic engineering (Menz et al., 2020). Compared to alternative genome editing methods, CRISPR offers superior speed, affordability, accuracy, and efficiency. The potential applications of CRISPR/Cas-mediated genome editing encompass diverse approaches that depend on the activities of Cas enzymes and the repair mechanisms for double-strand breaks (Huang et al., 2022). The CRISPR/Cas system has a broad spectrum of applications in gene function studies. CRISPR can induce gene silencing or knockout by inserting or deleting a few nucleotides, with subsequent repair through non-homologous end joining (Figure 1) (Peng et al., 2016). Alternatively, when homology-directed repair is established, CRISPR/Cas9 genome editing, alongside a DNA donor, can be utilized to facilitate the replacement of undesirable genes or to target overexpression of specific genes. Additionally, by rendering the Cas9 enzyme inactive and coupling it with transcription effectors or other enzymes (dCas9), the CRISPR/Cas system can be employed for epigenome editing and base editing. Most large-seeded legumes primarily rely on Agrobacterium-mediated transformation, though biolistic technologies have also been utilized (Table 4). Effective gene editing in grain legumes has been accomplished, with approximately 60% of successful edits out of multiple attempts using *Agrobacterium tumefaciens* C58 and *Agrobacterium rhizogenes* K599 as the delivery methods (Table 4).

Recent technological advancements have significantly broadened the capabilities of the CRISPR/Cas9 toolbox. The introduction of Cas9 nickase variants, such as dCas9 for CRISPR activation (CRISPRa) and CRISPR interference (CRISPRi), has allowed for greater precision in gene expression modulation (Piatek et al., 2015). Additionally, the development of D10A Cas9 allows for the introduction of targeted mutations without causing DNA breaks, making it suitable for base editing applications.

CRISPR/Cas9-mediated genome editing has been successfully implemented in certain grain legume crops (Table 4), including chickpea, soybean and cowpea, where transformation protocols are available. Several successful trials, optimizations and modifications have been reported. The following discussion covers publications addressing genes of interest, gene function and outcome of the CRISPR/Cas 9 mediated gene editing reported since 2015. First, in lentil, the scarcity of reports of successful transformation, and the transformation efficiency remains below 1%, despite attempts at *in vitro* plant regeneration using different lentil tissues, including epicotyls, nodal segments, cotyledonary nodes, shoot tips, embryonic axes and root structures. (Warkentin and McHughen, 1993; Akcay et al., 2009; Sarker et al., 2003; 2019). Of all the studies conducted, it was found that the cotyledon-attached decapitated embryo showed the most favorable response for *in vitro* regeneration after Agrobacterium-mediated genetic transformation (Sarker et al., 2003). Given that the quantity of shoots regenerated per explant significantly impacts the transformation efficiency and the effectiveness of CRISPR/Cas9-based gene editing, there is a need for future optimization of the protocol by using suitable mineral media and hormone combinations. Again, success in soybean trait transformation has been successful since the arrival of CRISPR/Cas9. For example, Liao et al. (2024) used the CRISPR/Cas9 system to target acyl-acyl carrier protein (ACP) thioesterases that are involved in fatty acid biosynthesis, resulting in mutations of GmFATA1 and GmFATA2, resulting in decreased fatty acid content and growth abnormalities. Alternatively, overexpression of ACP increased leaf fatty acid content, vegetative growth, seed yield, and seed fatty acid content. Additionally, Song et al. (2024) successfully edited the lincCG1 gene (a long non-coding RNA), which regulates soybean seed storage protein expression, generating β-conglycinin-deficient soybean lines with lower allergenicity.

Moreover, CRISPR/Cas9 is a powerful tool for enhancing disease resistance in pulse crops, but careful research is needed to minimize potential agronomic trade-offs. Singer et al. (2024) identified key target genes for improving resistance: knockout of *MLO*, *DMR6*, and *PMR4* strengthened defense against powdery mildew, downy mildew, and *Phytophthora* spp., respectively. Editing *EDR1*, *CRT1*, and *ERF* regulated disease-related signaling pathways, though some mutations impacted plant growth and drought tolerance. Disrupting transcription factors like *WRKY* and *JAZ2* reduced susceptibility to bacterial and fungal pathogens without affecting other agronomic traits. Modifications to *SWEET* gene promoters prevented pathogen exploitation of sugar transport while maintaining yield, and mutation of *PUB17* (E3 ubiquitin ligase) enhanced resistance to *Botrytis cinerea* and *Alternaria solani* in tomato. However, some edits caused unintended effects, such as reduced growth or increased susceptibility to other stressors, highlighting the need for careful evaluation before application in breeding programs.

Although CRISPR/Cas9 may provide results comparable to other gene editing techniques, some important challenges and limitations still exist. One significant challenge is the issue of off-target effects that occur when CRISPR/Cas9 inadvertently alters DNA sequences that are similar to the intended target. This can lead to unintended genetic changes and raise concerns about the safety and accuracy of the technique. Gene editing, especially through the CRISPR/Cas9 system, holds great promise for developing new plant varieties, but the diverse regulatory landscape across jurisdictions poses challenges and trade dilemmas for plant breeders (Singer et al., 2024). Regulatory frameworks for gene editing vary significantly across countries, and ongoing debates regarding their responsible use contribute to global uncertainty in technology adoption (Peters, 2021). This regulatory ambiguity has direct implications for food security, as it influences the pace at which gene-edited crops—designed to be more resilient, nutritious, and high-yielding—can be developed and distributed. In countries such as the United States and Canada, regulatory decisions are based on the novelty of the trait rather than the technology used, enabling a more streamlined path for deploying genome-edited crops. For instance, the U.S. classifies gene-edited varieties without foreign DNA as conventionally bred, while Canada applies a risk-based, product-triggered approach that assesses the safety of new characteristics, regardless of the breeding method (Table 3). Argentina has similarly positioned itself as a global leader by implementing early and innovation-friendly policies that exempt non-transgenic edits from GMO regulation, accelerating access to improved crop varieties (Rose et al., 2020; Peters, 2021) (Table 2). Such regulatory flexibility is crucial for ensuring that gene editing can effectively contribute to global food security by enabling the rapid development of crops that address malnutrition, climate stress, and yield limitations.

**TABLE 3:** Comparative overview of global regulatory frameworks for gene editing for food security.

Country/Region	Regulatory approach	GMO classification	Description
United States	Trait-based, case-by-case	Not a GMO if no foreign DNA	USDA (via SECURE Rule) exempts certain CRISPR crops if indistinguishable from conventionally bred varieties. FDA review is voluntary unless food safety issues arise
European Union	Process-based, precautionary	Always a GMO	All gene-edited organisms fall under 2001/18 GMO Directive, regardless of whether transgenes are present
China	Revised 2022, progressive	Not a GMO if no transgenes	Introduced new guidelines for approval of gene-edited crops; streamlined approval process for CRISPR-edited plants without foreign DNA.
Argentina	Product-based	Not a GMO if no new genetic combination	First country to implement case-by-case regulation of genome-edited crops through Resolution 173/2015
Brazil	Product-based	Not a GMO if mimicking conventional breeding	CTNBio (National Technical Commission on Biosafety) regulations allow CRISPR if no recombinant DNA remains
Japan	Tiered approach	Not a GMO if no transgene	MHLW and MAFF exempt CRISPR-edited products without transgenes from strict GMO law; GABA tomato approved in 2021
Canada	Novel trait–based	It depends on trait novelty, not method	Product-based trigger: gene-edited crops may require full risk assessment if traits are novel
Australia	Product-based	Not a GMO for SDN-1 edits	As of 2019, SDN-1 gene edits (no template, no transgene) are excluded from GMO regulation

Sources: Smyth (2020), Mbaya et al. (2022), and Liberty et al. (2024).

### Prime editing: a precise “search and replace” genome editing technique

Achieving precise genome editing has been a central goal for applications in functional genomics and crop enhancement. In comparison to homology-directed repair (HDR), base editing techniques offer significantly greater efficiency—up to 100 times higher—in producing targeted mutations (Komor et al., 2016; Mishra et al., 2020). Among base editors, cytosine base editors (CBEs) and adenine base editors (ABEs) are widely utilized, enabling C⋅G-to-T⋅A and A⋅T-to-G⋅C conversions, respectively, through the integration of nCas9 or dCas9 with specific deaminases. These base editors have been successfully utilized in a variety of crops, including rice, wheat, maize, tomatoes, and cotton (Nishida et al., 2016; Shimatani et al., 2017; Zong et al., 2017; Kang et al., 2018; Li et al., 2018; Qin et al., 2020). While base editors have demonstrated remarkable efficiency in plants, they are currently restricted to facilitating only four specific types of base modifications. To effectively manipulate a broader range of agronomic traits, it is essential to achieve the remaining eight nucleotide substitutions (A⋅T-to-C⋅G, C⋅G-to-A⋅T, T⋅A-to-A⋅T, and G⋅C-to-C⋅G) as well as induce deletions or insertions (Biswas et al., 2022a).

Prime editing, a recently developed method derived from CRISPR/Cas9 technology, combines a modified reverse transcriptase paired with a catalytically inactive 8Cas9 endonuclease (nCas9), and a prime editing guide RNA to facilitate precise genetic modifications (Mishra et al., 2020; Biswas et al., 2022a; Zhong et al., 2022). Prime editing provides a compelling solution by enabling highly precise and versatile genome modifications, allowing nearly any desired type of edit to be made. This approach utilizes three plant prime editor (PPE) systems: PPE2, PPE3, and PPE3b. This system combines an nCas9 (H840A) fused with an engineered M-MLV reverse transcriptase and a prime editing guide RNA (pegRNA), which includes a primer binding site (PBS) and a reverse transcriptase template. Additionally, PPE3 and PPE3b incorporate a nicking single guide RNA (sgRNA) to enhance DNA repair efficiency (Anzalone et al., 2019; Biswas et al., 2022a). Among these, PPE3b stands out by producing fewer insertions or deletions (indels) in plant cells compared to mammalian cells, even though the editing efficiencies of the three systems are comparable (Anzalone et al., 2019). Once optimized for specific crops, prime editing holds tremendous potential in achieving a broader range of precise mutations, thereby contributing significantly to crop improvement efforts (Tang et al., 2020).Recent research has shown the successful implementation of prime editing in several plant species, including rice, wheat, maize, potato, and tomato (Food and Agriculture Organization, 2018; Lin et al., 2020; Tang et al., 2020; Veillet et al., 2020; Lu et al., 2021). In wheat, single nucleotide substitutions such as A-to-T, C-to-G, G-to-C, T-to-G, and C-to-A were achieved with frequencies as high as 1.4% (Lin et al., 2020). Remarkably, in rice, the use of dual prime editing guide RNAs (pegRNAs) along with an optimized primer binding site (PBS) significantly increased editing efficiency, achieving rates of up to 17% (Lin et al., 2021). However, there is room for further improvement in prime editing efficiency across different crops due to several challenges. One major factor is the low editing efficiency in many plant species, which can be attributed to suboptimal delivery methods and plant-specific DNA repair mechanisms that do not always favor precise edits. Additionally, plant regeneration from edited cells is often difficult in many crop species, limiting the recovery of successfully edited plants (Baloglu, et al., 2022). Plant protoplasts have been identified as effective platforms for refining gene editing techniques primarily because they lack a rigid cell wall, which simplifies the delivery of gene editing components like nucleases, guide RNAs, and donor templates. This wall-free cell environment allows direct access to the cellular machinery, enhancing the efficiency of component uptake. Moreover, protoplasts enable controlled experimentation on DNA repair pathways and editing accuracy, providing valuable a deeper understanding of the mechanisms and outcomes of genome editing in a simplified and manipulable setting (Petersen et al., 2019; Biswas et al., 2022b; Lin et al., 2021). While prime editing in legumes is still underexplored, its demonstrated success in rice and other crops suggests that this advanced technique could be effectively implemented in grain legumes as well. A recent study advanced the optimization of prime editing by targeting mutant GFP in protoplasts derived from rice, peanuts, chickpeas, and cowpeas, leveraging transient expression systems to refine editing parameters. The study successfully obtained edited mutant GFP protoplasts in peanuts, chickpeas, and cowpeas after transformation with dual pegRNA vectors, although the editing efficiency was relatively low compared to rice, ranging from 0.2% to 0.5% (Biswas et al., 2022a). These initial findings offer promising prospects for integrating prime editing into legume breeding programs that can help speed up crop improvement. Despite the existence of only a single publication showcasing prime editing in legume crops, it is clear that this technique can be successfully applied to peanuts, cowpeas, and chickpeas. As further advancements and optimizations are made, these findings have the potential to enable more accurate editing of essential traits in legume crops, paving the way for addressing future challenges and enhancing food security.

### Scientometric analysis of CRISPR/Cas9 application in grain legumes

**FIGURE 2:**
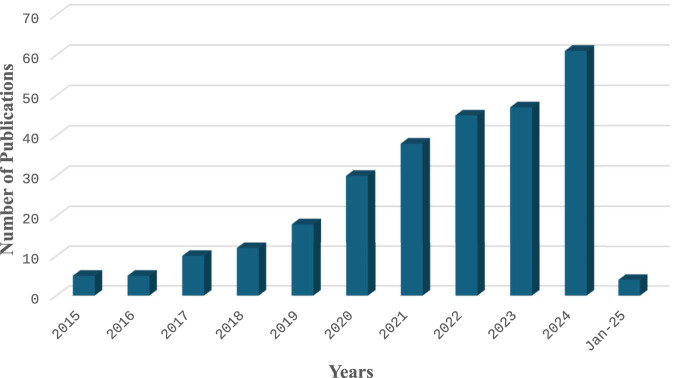
Number of publications on CRISPR/Cas9 applications in grain legumes from 2015 to 2024, based on the Web of Science Database.

Literature retrieved from WoS revealed that the CRISPR/Cas 9 application began in 2015 as seen in Figure 2. The first published study was conducted by Jacob, who analyzed the hydro-ecology of the fen system at Leiper Posse in eastern Germany.

Zhang, et al. (2014) performed gene editing in legumes, and Jacobs et al. (2015) performed the first targeted gene modification utilizing CRISPR/Cas9 gene editing in grain legumes in 2015. In 2024, the number of publications reached 61, reflecting a notable increase in research activity. Much of this research was particularly prominent in China, highlighting the country’s leading role in advancing CRISPR/Cas9 applications in grain legumes.

In Figure 2, we present a visual representation of the number of publications about grain legumes from the year 2015 to September 2024, utilizing data sourced from the Web of Science database. The figure provides a comprehensive overview of the scholarly output in the field of grain legumes during this timeframe, highlighting the growth and trends in research activity within this domain.

**FIGURE 3:**
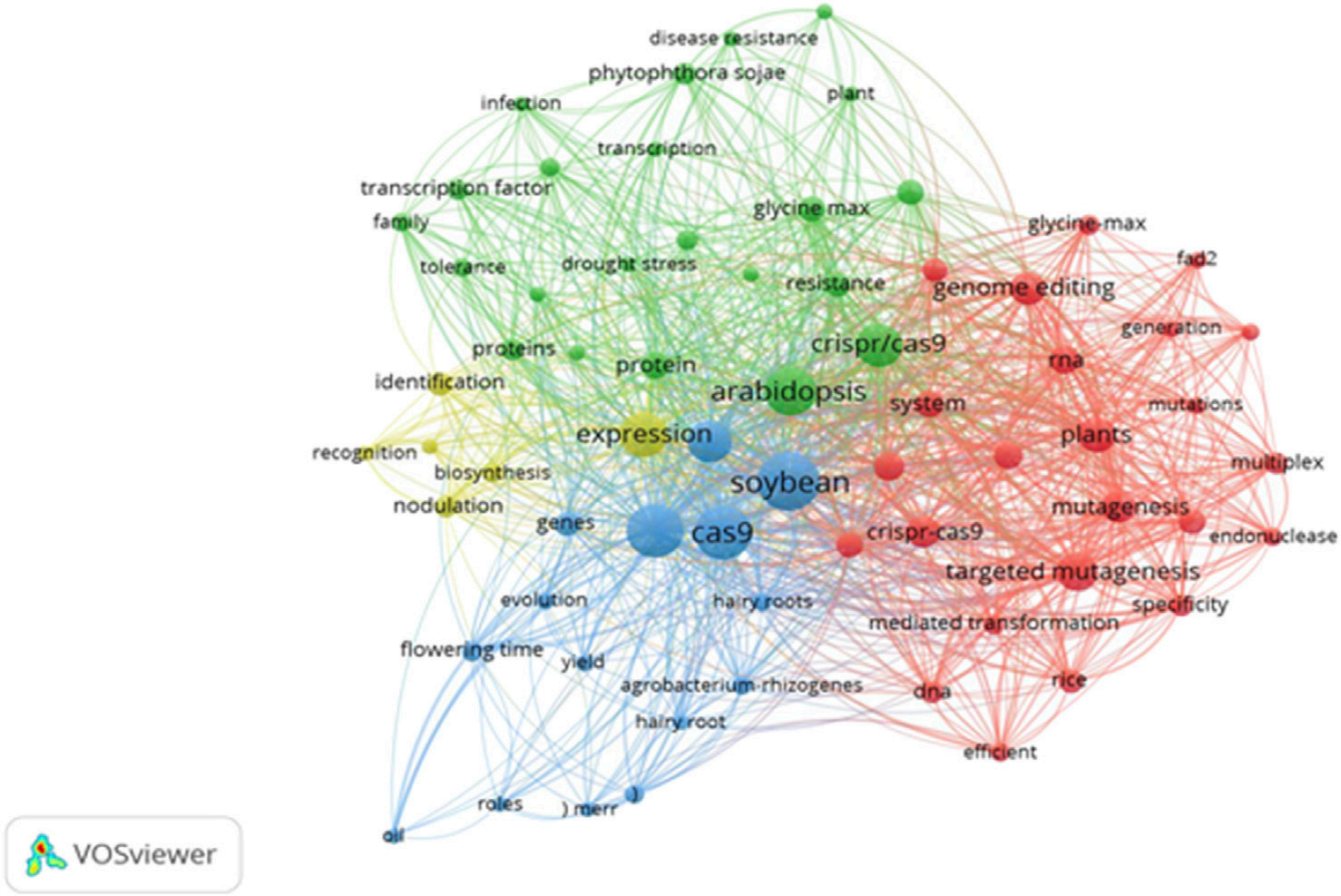
Network visualization.

Figure 3 offers a network visualization of the key terms and concepts associated with gene editing in grain legumes. The nodes represent frequently occurring terms in titles, abstracts, and keywords, grouped into distinct clusters based on co-occurrence. The red cluster focuses on gene-editing technologies, particularly CRISPR/Cas9 and its applications in plants like soybean and Arabidopsis. The green cluster centers on stress tolerance, highlighting research aimed at improving grain legume resilience against environmental challenges such as drought (Figure 3). The blue cluster includes fundamental studies on gene expression and protein identification in legumes (Figure 3). The map underscores the diversity of research topics, with interconnected clusters showing how different areas like biotechnology, stress resistance, and crop improvement are tightly linked. The prominence of soybeans (*Glycine max*) as a large circle highlights its significance as a major grain legume in gene-editing research, predominantly with CRISPR/Cas9 technology. The large size of the circle indicates that soybeans are extensively studied, making them a central focus of research aimed at improving traits like yield, disease resistance, and environmental adaptability (Figure 3).

Bibliographic coupling at the country level: showcasing global collaboration patterns in grain legume research (Supplementary Figure S1). Countries are represented by nodes, with larger nodes indicating greater research output. Although there are few countries showing successful research in CRISPR application in legumes, China stands out as the most significant contributor, reflecting its central role in this field. Strong connections between China and countries like Canada and Australia suggest robust international collaboration. The map highlights distinct regional clusters, such as East Asia (China, Japan, South Korea) and Western countries (Canada, Australia), indicating focused yet interconnected research networks. This geographic distribution shows how key nations are shaping advancements in gene editing for food security.

The researcher-level bibliographic coupling map identifies key influencers and collaborative groups in the field. The network features nodes representing researchers, with larger nodes indicating highly prolific or influential figures, such as Chen Li and Hou Wen Sheng (Supplementary Figures S1, S2). Their research predominantly involves two key legumes: soybean and peanut (groundnut) (Table 4). Their work focuses on applying CRISPR/Cas9 and similar gene-editing technologies are utilized to enhance these crops. The dense connections within the red cluster reveal a tightly knit group of researchers actively driving innovation in gene editing technologies with CRISPA/Cas9. Smaller clusters, like the green group, may represent more specialized or emerging areas of research, such as disease resistance. This map highlights the importance of collaboration among leading scholars and sheds light on the key contributors advancing gene editing in grain legumes.

**TABLE 4:** CRISPR/Cas9 - mediated genome editing applications in grain legumes.

Grain legume	Delivery method	Gene(s) of interest	Gene function/trait	Outcome	References
*Cicer arietinum* (Chickpea)	Agrobacterium-mediated delivery of a codon-optimized CRISPR/Cas9 system	Target genes in chickpea (specific genes not detailed)	Optimize protocol for targeted mutation	Researchers successfully developed an optimized CRISPR/Cas9 system designed for chickpeas, facilitating precise targeted mutagenesis	Gupta, et al. (2023)
*Vigna unguiculata* (Cowpea)	CRISPR/Cas9 system delivered through *Agrobacterium rhizogenes*-mediated hairy root transformation	*SYMRK* (Symbiosis Receptor-Like Kinase)	Symbiotic nitrogen fixation genes	Disrupted *SYMRK* gene inhibits the formation of nodules	Ji et al. (2019)
*Cicer aritinum* (Chickpeas)	A polyethylene glycol (PEG4000)-mediated	4-coumarate ligase) and Reveille 7	4CL is involved in the phenylpropanoid pathway, contributing to lignin biosynthesis and plant defense, while RVE7 is a MYB transcription factor that regulates the plant’s circadian rhythm	The study achieved high efficiency editing for the RVE7 gene *in vivo*, while the 4CL gene showed lower editing efficiency	Badhan et al. (2021)
*Cicer arietinum* (Chickpea) *and Lens culinaris* (Lentils)	CRISPR/Cas9 through biolistic particle delivery	Drought-tolerance related genes (e.g., *DREB1A*)	Enhancing drought tolerance	Generated mutant lines showed significant improvements in drought tolerance, demonstrating the feasibility of CRISPR/Cas9 in chickpea and lentil breeding	Roy and Sandhu (2024)
*Arachis hypogaea* (peanut)	*A. rhizogenes* – mediated delivery	*ahFAD2A* and *ahFAD2B* (Fatty Acid Desaturase genes)	Encoding enzymes that convert oleic acid into linoleic acid	Mutations in the *ahFAD2A* and *ahFAD2B* genes resulting in high oleic acid in peanut	Yuan et al. (2019)
*Arachis hypogaea* (Peanut)	CRISPR/Cas9 system via *A bacterium hairy* root transformation system	*AhNFR1* and *AhNFR5* (Nod Factor Receptors)	Formation of root nodules for nitrogen fixation	Knockout mutants of *AhNFR1* and *AhNFR5* genes in peanut hairy roots: Mutants with edited *AhNFR5* genes showed a non-nodulating	Shu et al. (2020)
*Arachis hypogaea* (Peanut)	*PEG*-mediated protoplast transformation	*Ara h 2*	Major allergen	Targeting *Ara h 2* and validated their efficiency through *in vitro* digestion. Deep sequencing revealed indel mutations ranging from 0.13% to 0.8%, disrupting the protein sequence	Biswas et al. (2022b)
*Vigna unguiculata* L. (Cowpea)	Agrobacterium-mediated infiltration	*VuSPO11-1*	Cowpea meiosis gene involved in reproductive development	Male and female sterilities	Juranić et al., 2020
				Achieved 68.6% editing efficiency	Che et al. (2021)
*Phaseolus vulgaris* (Common Bean)	CRISPR/Cas9 Gene Editing	*PvAPRT1* and *PvAPRT5* (Adenine Phosphoribosyl Transferase genes)	Regulation of purine nucleotide salvage and cytokinin homeostasis	*PvAPRT1* primarily functions in adenine salvage, while *PvAPRT5* regulates cytokinin levels, affecting root and nodule development	López et al. (2025)
*Lathyrus sativus* (Grass pea)	Hairy root transformation and CRISPR gene editing	*LsOCS* (Oxalyl-CoA synthetase)	Key enzyme in oxalate metabolism and linked to β-ODAP production	Editing *LsOCS* led to higher oxalate accumulation but did not alter β-ODAP levels; complementation in Arabidopsis restored normal oxalate levels and seed coat integrity	Verma et al. (2024)
*Glycine max* (Soybean)	*Agrobacterium rhizogenes*	DD20, DD43, ALS1	Genomic targets; herbicide resistance (ALS1)	Achieved targeted mutagenesis at DD20 and DD43 loci with mutation frequencies of 59% and 76%, respectively.	Li et al. (2015)
*Glycine max* (Soybean)	Agrobacterium-mediated delivery	Green Fluorescent Protein (GFP) Transgene and 9 indigenous Loci modification	Fluorescent protein	The study found that 95% of the hairy-root transgenic examined showed targeted mutations in DNA, with bi-allelic mutations in eight of the nine targeted loci	Jacobs et al. (2015)
*Glycine max* (Soybeans)	Agrobacterium rhizogenes-mediated hairy root transformation	Single-copy soybean gene, *Glyma07g14530*	A putative glucosyl-transferase	Achieved mono- and bi-allelic modifications; mutation efficiencies ranged from 14.7% to 20.2%	Sun et al. (2015)
		*Glyma12g37050*	Ethylene signaling	Biallelic mutations of *Glyma08g02290* and Glyma*06g14180* were found in transgenic hairy roots Off-target activities were linked to Glyma12g37050 and Glyma06g14180 and were also reported	
		*Glyma06g14180*	Uncharacterized protein		
		*Glyma08g02290*	Potassium ion transporter gene		
*Glycine max* (Soybeans)	Agrobacterium-mediated delivery	*GmFT2a (Glyma16g26660)*	Flowering Locus T homolog in soybean: for flowering time regulation	Ft2a mutants with a 1618 bp deletion exhibited late-flowering phenotypes	Cai et al. (2018a)
*Glycine max* (Soybeans)	Agrobacterium-mediated delivery	*GmFT5a (Glyma16g04830)*	The *FEI 2* gene encode a leucine-rich repeat (LRR) receptor-like kinase that functions as a serine/threonine protein kinase	Deletions ranging from 3.6 kb to 10.4 kb were successfully achieved; demonstrated feasibility of deleting large DNA fragments in soybean genome	Cai et al. (2018b)
*Glycine max* (Soybeans)	Agrobacterium-mediated delivery	*GmPDS11* and *GmPDS18*	Coding for phytoene dehydrogenase/chromoplastic protein	development of albino and stunted buds	Curtin et al. (2018)
*Glycine max* (Soybeans)	*Agrobacterium tumefaciens-mediated* delivery	*GmFT2a* and *GmFT5a*	Regulating flowering time	Loss-of-function due to deletion mutations	Cai et al. (2019)
*Glycine max* (Soybeans)	*A. tumefaciens*- mediated delivery	GmSPL9a, GmSPL9b, GmSPL9c, GmSPL9d	Regulation of plant architecture	Mutagenesis of GmSPL9 - Altered plant architecture	Bao et al. (2019)
*Glycine max* (Soybeans)	*A. tumafacien*-mediated delivery	FAD2-2 microsomal omega-6 desaturase	Fatty acid composition	Precise alteration of the FAD2-2 gene to lower omega-6 fatty acid levels	Al Amin et al. (2019)
*Glycine max (Soybeans)*	*A. rhizogenes* - mediated delivery with small guide RNA *(sg*RNA)	Glyma03g36470	Eukaryotic translation initiation factor	The mutation efficiency ranged from 2.8% to 20.6%, with the GmU6-8 (20.3%) and GmU6-10 (20.6%) promoters yielding the highest mutation efficiencies. The five showed high transcriptional activity	Di et al. (2019)
		Glyma14g04180	Late-embryogenesis abundant (LEA) proteins		
		Glyma06g136900	Uncharacterized protein		
*Glycine max* (Soybeans)	CRISPR/Cas9 system	*GmFEI2* and *GmSHR* and bar (a transgene)	Root development and resistance	Induce mutations in the target genes (both endogenous and exogenous) within soybean hairy roots	Cai et al. (2015)
*Glycine max* (Soybeans)	CRISPR/Cas9 system	Glycinin (11S) and Conglycinin (7S): The specific genes targeted include Glyma.20g148400, Glyma.03g163500, and Glyma.19g16490	Genes encoding essential proteins for nutrient storage and seed development	Alterations occurred in three of the nine distinct storage protein genes. Mutation efficiency ranged from 3.8% to 43.7% depending on the gene, with Glyma.19g164900 showing the highest editing efficiency.	Li et al. (2019)
*Glycine max* (Soybeans)	CRISPR/Cas9 system *via Agrobacterium-mediated transformation*	E1 gene (Glyma.06G207800)	Major gene controlling photoperiod sensitivity	Early flowering	Han et al. (2019)
*Glycine max* (Soybeans)	CRISPR/Cas9 system via *A. tumefaciens*-– mediated delivery	Seed lipoxygenase genes: *GmLox1*, *GmLox2*, *GmLox3*	Production of lipoxygenase enzymes, which are associated with undesirable flavors in soybean	Free of seed lipoxygenase: resulted in soybean seeds with improved taste quality due to the absence of the off-flavoring enzymes	Wang et al. (2020)
*Glycine max* (Soybeans)	CRISPR/Cas9 using egg cell-specific promoters	Validating genome editing systems rather than specific genes	Focus is on improving the efficiency and heritability of gene edits in plant	CaMV 2 × 35S promoter in hairy roots showed high somatic mutation frequencies	Zheng et al. (2020)
*Glycine max* (Soybeans)	*A. tumefaciens*– mediated delivery	*GmFT2a* and *GmFT5a*	flowering time control gene	Successful development of *GmFT2a, GmFT5a*, and *GmFT2a GmFT5a* mutants	Cai et al. (2020b)
*Glycine max* (Soybeans)	*A. tumefaciens*– mediated delivery	*GmFT2a*	Regulation of flowering time	C to G and C to T base substitutions; delayed flowering observed in homozygous mutants	Cai et al. (2020a)
		*GmFT4*		C to G base substitution; chimeric mutants generated but no heritable changes observed	
*Glycine max* (Soybeans)	CRISPR/Cas9 system using a pooled transformation approach	Targeted a total of 102 potential genes along with their paralogs	Regulating nodule numbers: essential for nitrogen fixation	Multiplex mutagenesis (double mutant lines with increased nodulation	Bai et al. (2020)
*Glycine max* (Soybeans)	*A. rhizogenes* – mediated delivery	β-ketoacyl- [acyl carrier protein] synthase 1 (KASI)	Conversion of sucrose to oil in plants	Mutations in the KASI gene, such as increased seed sucrose content, decreased seed oil content, and wrinkled seed appearance	Virdi, et al. (2020)
*Glycine max* (Soybeans)	*Agrobacterium*	*GmFAD2-1A, GmFAD2-2A*	Responsible for converting oleic acid into linoleic acid	Increase in oleic acid content and a decrease in linoleic acid content	Wu et al. (2020)
*Glycine max* (Soybeans)	CRISPR/Cas9 system	CRISPR/Cas9 gene editing	Acyl-acyl carrier protein (ACP) thioesterases are involved in fatty acid (FA) biosynthesis	Mutation of *GmFATA1* or *GmFATA2* led to reduced leaf FA content and growth defects; overexpression increased leaf FA content, vegetative growth, seed yield, and seed FA content	Liao et al. (2024)
*Glycine max* (Soybeans)	CRISPR/Cas9 system	LincCG1 (lincRNA)	Regulates expression of soybean seed storage protein (SSP) genes; associated with β-conglycinin, a major allergen	Generated β- conglycinin-deficient soybeans lines with lower allergenicity and higher nutritional value	Song et al. (2024)
*Glycine max* (Soybeans)	Agrobacterium-mediated transformation	*cle1a/2a* (*ric1a/2a*)	Regulates nodulation and carbon distribution	Increased nodules in a controlled manner, improved grain yield, higher protein levels, and stable oil content	Zhong et al. (2024)
*Glycine max* (Soybeans)	Agrobacterium-mediated transformation	*GRF3-GIF1* chimera	Enhances regeneration and transformation efficiency	Increased transformation efficiency across multiple genotypes (up to 23.95%); compatible with CRISPR/Cas9 for improved gene editing	Zhao et al. (2025)
*Glycine max* (Soybeans)	DNA-free CRISPR/Cas9 (Ribonucleoprotein-based)	*GmBAS1* and *GmBAS2* (ß-amyrin synthase genes)	Involved in soyasaponin biosynthesis, affecting soybean seed taste	*GmBAS1* plays a key role in soyasaponin production; its targeted mutation led to the elimination of soyasaponins in seeds, roots, stems, and leaves	Asa et al. (2025)
*Glycine max* (Soybeans)	CRISPR/Cas9 genome editing	*GmDWF1a* and *GmDWF1b* (Brassinosteroid biosynthesis genes)	Involved in brassinosteroid production, influencing plant height and yield	Mutations in *GmDWF1a* and *GmDWF1b* caused reduced plant height; single mutants exhibited mild dwarfism, while double mutants showed more pronounced height reduction; pod production increased in *GmDWF1a* mutants; application of brassinolide restored normal height	Xiang et al. (2024)
*Glycine max* (Soybeans)	CRISPR/Cas9 genome editing	*GmARM* (Stress-related gene)	Regulates response to abiotic and biotic stress, including salt, alkali, and Phytophthora root rot resistance	*GmARM* mutants showed improved survival under salt and alkali stress, as well as increased resistance to *Phytophthora* infection; altered expression of stress-related genes, including those in the ABA and salicylic acid signaling pathways; no significant agronomic abnormalities in gene-edited plants	Luo et al. (2024)
*Glycine max* (Soybeans)	CRISPR/Cas9 genome editing	*GmFLA22a* (Fasciclin-like arabinogalactan protein)	Plays a role in anther and pollen development, affecting male fertility	Mutant plants had a lower seed-setting rate despite normal pollen viability; delayed pollen release and thickened locule walls were observed in anthers	Cao et al. (2024)

## Conclusion

Grain legumes have long been a cornerstone of global nutrition and agriculture, providing essential proteins, micronutrients, and environmental benefits. Their role extends far beyond their nutritional value, contributing to soil health, reducing reliance on chemical fertilizers, and enhancing resilience to climate change. These qualities make legumes critical to achieving global food security and addressing the challenges posed by a growing population and environmental degradation. The advent of advanced gene-editing technologies, including CRISPR/Cas9, TALENs, ZFNs, and Prime Editing, has revolutionized the field of crop improvement. This review highlights how these tools have significantly advanced our ability to enhance legume agronomic traits, including productivity, resilience to stress, and nutritional composition. Among these, CRISPR/Cas9 has revolutionized genetic engineering with its high precision, efficiency and adaptability, enabling breakthroughs such as climate-resilient soybean varieties and allergen-reduced crops. Prime Editing, although still in its infancy for legumes, holds immense potential to achieve even more precise genetic modifications. Despite these advancements, the implementation of gene editing in legumes faces challenges, including regulatory uncertainties, technological limitations, and ethical concerns. Addressing these barriers will require harmonized global regulations, robust international collaboration, and investments in research and capacity-building, particularly in developing nations where food insecurity is most acute. By leveraging these technologies and fostering global partnerships, we can unlock the full potential of legumes, paving the way for a more sustainable, equitable, and resilient agricultural future. The continued evolution of gene editing, coupled with a commitment to inclusivity and innovation, promises to transform legumes into even more vital components of global food systems, securing nutrition and sustainability for generations to come.

## References

[B1] Ahmad A. Zhong C. Wang W. Zhang Y. (2021). Advances in plant genome editing: methods, applications, and future perspectives. Front. Genet. 12, 690481. 10.3389/fgene.2021.690481

[B2] Akcay U. C. Mahmoudian M. Kamci H. Yucel M. Oktem H. A. (2009). Agrobacterium tumefaciens‐ mediated genetic transformation of a recalcitrant grain legume, lentil (*Lens culinaris Medik*). Plant Cell Rep. 28, 407–417. 10.1007/s00299-008-0652-4 19083242

[B3] Al Amin N. Ahmad N. Wu N. Pu X. Ma T. Du Y. (2019). CRISPR-Cas9 mediated targeted disruption of FAD2–2 microsomal omega-6 desaturase in soybean (Glycine max. L). BMC Biotechnol. 19 (1), 9–10. 10.1186/s12896-019-0501-2 30691438 PMC6350355

[B4] Anzalone A. V. Randolph P. B. Davis J. R. Sousa A. A. Koblan L. W. Levy J. M. (2019). Search-and-replace genome editing without double-strand breaks or donor DNA. Nature 576 (7785), 149–157. 10.1038/s41586-019-1711-4 31634902 PMC6907074

[B5] Arrighi J. F. Barre A. Ben Amor B. Bersoult A. Soriano L. C. Mirabella R. (2006). The Medicago truncatula lysin [corrected] motif-receptor-like kinase gene family includes NFP and new nodule-expressed genes. Plant physiol. 142 (1), 265–279. 10.1104/pp.106.084657 16844829 PMC1557615

[B6] Asa H. Kuwabara C. Matsumoto K. Shigeta R. Yamamoto T. Masuda Y. (2025). Simultaneous site-directed mutagenesis for soybean ß-amyrin synthase genes via DNA-free CRISPR/Cas9 system using a single gRNA. Plant Cell Rep. 44, 40. 10.1007/s00299-025-03433-w 39873837

[B7] Asare A. T. Gowda B. S. Galyuon I. K. Aboagye L. M. Takrama J. F. Timko M. P. (2010). Assessment of the genetic diversity in cowpea (Vigna unguiculata L. Walp.) germplasm from Ghana using simple sequence repeat markers. Plant Genetic Resources, 8, 142–150. 10.1017/S1479262110000092

[B8] Asiamah J. Y. (2020). Phenotypic and molecular characterization of *Striga gesnerioides* resistance among cowpea (Vigna unguiculata L. Walp.) breeding lines Master’s thesis. University of Cape Coast, Cape Coast Ghana. University of Cape Coast Institutional Repository. Available online at: https://ir.ucc.edu.gh/xmlui/bitstream/handle/123456789/7674/ASIAMAH%2C%202020%201.pdf?sequence=1.

[B9] Badhan S. Ball A. S. Mantri N. (2021). First report of CRISPR/Cas9 mediated DNA-free editing of *4CL* and *RVE7* genes in chickpea protoplasts. Int. J. Mol. Sci. 22 (1), 396. 10.3390/ijms22010396 33401455 PMC7795094

[B10] Bai M. Yuan J. Kuang H. Gong P. Li S. Zhang Z. (2020). Generation of a multiplex mutagenesis population via pooled CRISPR‐Cas9 in soya bean. Plant Biotechnol. J. 18 (3), 721–731. 10.1111/pbi.13239 31452351 PMC7004907

[B11] Baloglu M. C. Celik Altunoglu Y. Baloglu P. Yildiz A. B. Türkölmez N. Özden Çiftçi Y. (2022). Gene-editing technologies and applications in legumes: progress, evolution, and future prospects. Front. Genet. 13, 859437. 10.3389/fgene.2022.859437 35836569 PMC9275826

[B12] Bao A. Chen H. Chen L. Chen S. Hao Q. Guo W. (2019). CRISPR/Cas9-mediated targeted mutagenesis of GmSPL9 genes alters plant architecture in soybean. BMC Plant Biol. 19, 131. 10.1186/s12870-019-1746-6 30961525 PMC6454688

[B13] Bedell V. M. Wang Y. Campbell J. M. Poshusta T. L. Starker C. G. Krug II R. G. (2012). *In vivo* genome editing using a high-efficiency TALEN system. Nature 491 (7422), 114–118. 10.1038/nature11537 23000899 PMC3491146

[B14] Berglund-Brücher O. Brücher H. (1976). The South American wild bean (Phaseolus aborigineus Burk.) as ancestor of the common bean. Econ. Bot. 30, 257–272. 10.1007/bf02909734

[B15] Biswas S. Bridgeland A. Irum S. Thomson M. J. Septiningsih E. M. (2022a). Optimization of prime editing in rice, peanut, chickpea, and cowpea protoplasts by restoration of GFP activity. Int. J. Mol. Sci. 23 (17), 9809. 10.3390/ijms23179809 36077206 PMC9456013

[B16] Biswas S. Wahl N. J. Thomson M. J. Cason J. M. McCutchen B. F. Septiningsih E. M. (2022b). Optimization of protoplast isolation and transformation for a pilot study of genome editing in peanut by targeting the allergen gene Ara h 2. Int. J. Mol. Sci. 23 (2), 837. 10.3390/ijms23020837 35055026 PMC8775966

[B17] Boch J. Scholze H. Schornack S. Landgraf A. Hahn S. Kay S. (2009). Breaking the code of DNA binding specificity of TAL-type III effectors. Science 326 (5959), 1509–1512. 10.1126/science.1178811 19933107

[B18] Brabin B. J. Coulter J. B. (2009). “Nutrition-associated disease,” in Manson's tropical diseases (WB Saunders), 537–555. 10.1016/B978-1-4160-4470-3.50034-3

[B19] Cai Y. Chen L. Liu X. Guo C. Sun S. Wu C. (2018a). CRISPR/Cas9-mediated targeted mutagenesis of GmFT2a delays flowering time in soybean. Plant Biotechnol. J. 16 (2), 176–185​. 10.1111/pbi.12855 28509421 PMC5785355

[B20] Cai Y. Chen L. Liu X. Sun S. Wu C. Jiang B. (2015). CRISPR/Cas9-mediated genome editing in soybean hairy roots. PLoS One 10 (8), e0136064. 10.1371/journal.pone.0136064 26284791 PMC4540462

[B21] Cai Y. Chen L. Sun S. Wu C. Yao W. Jiang B. (2018b). CRISPR/Cas9-mediated deletion of large genomic fragments in soybean. Int. J. Mol. Sci. 19 (12), 3835. 10.3390/ijms19123835 30513774 PMC6321276

[B22] Cai Y. Chen L. Zhang Y. Yuan S. Su Q. Sun S. (2020a). Target base editing in soybean using a modified CRISPR/Cas9 system. Plant Biotechnol. J. 18 (10), 1996–1998. 10.1111/pbi.13386 32311214 PMC7540304

[B23] Cai Y. Chen L. Zhang Y. Yuan S. Su Q. Sun S. (2019). Mutagenesis of *GmFT2a* and *GmFT5a* mediated by CRISPR/Cas9 contributes to the study of flowering time regulation in soybean. Int. J. Mol. Sci. 20 (3), 540. 10.3390/ijms20030540 30696013 PMC6387248

[B24] Cai Y. Wang L. Chen L. Wu T. Liu L. Sun S. (2020b). Mutagenesis of GmFT2a and GmFT5a mediated by CRISPR/Cas9 contributes for expanding the regional adaptability of soybean. Plant Biotechnol. J. 18 (2), 298–309. (MDPI). 10.1111/pbi.13199 31240772 PMC6920152

[B25] Cao Z.-L. Li J.-H. Zhou M.-H. Zhang M.-T. Wang N. Chen Y.-F. (2024). Functional analysis of *GmFLA22a* in soybean male fertility. Yi Chuan 46 (4), 1–12. 10.16288/j.yczz.24-030 38632095

[B26] Che P. Chang S. Simon M. K. Zhang Z. Shaharyar A. Ourada J. (2021). Developing a rapid and highly efficient cowpea regeneration, transformation and genome editing system using embryonic axis explants. Plant J. 106 (3), 817–830. 10.1111/tpj.15202 33595147 PMC8252785

[B27] Curtin S. J. Xiong Y. Michno J. M. Campbell B. W. Stec A. O. Čermák T. (2018). CRISPR/Cas9 and TALENs generate heritable mutations for genes involved in small RNA processing of *Glycine max* and *Medicago truncatula* . Plant Biotechnol. J. 16 (6), 1125–1137. 10.1111/pbi.12857 29087011 PMC5978873

[B28] Curtin S. J. Zhang F. Sander J. D. Haun W. J. Starker C. Baltes N. J. (2011). Targeted mutagenesis of duplicated genes in soybean with zinc-finger nucleases. Plant physiol. 156 (2), 466–473. 10.1104/pp.111.172981 21464476 PMC3177250

[B29] Dave K. Kumar A. Dave N. Jain M. Dhanda P. S. Yadav A. (2024). Climate change impacts on legume physiology and ecosystem dynamics: a multifaceted perspective. Sustainability 16 (14), 6026. 10.3390/su16146026

[B30] De L. C. De T. (2019). Healthy food for healthy life. J. Glob. Biosci. 8, 6453–6468. Available online at: https://www.researchgate.net/publication/336231128_HEALTHY_FOOD_FOR_HEALTHY_LIFE.

[B31] Demorest Z. L. Coffman A. Baltes N. J. Stoddard T. J. Clasen B. M. Luo S. (2016). Direct stacking of sequence-specific nuclease-induced mutations to produce high oleic and low linolenic soybean oil. BMC plant Biol. 16, 225–228. 10.1186/s12870-016-0906-1 27733139 PMC5062912

[B32] Di Y. H. Sun X. J. Hu Z. Jiang Q. Y. Song G. H. Zhang B. (2019). Enhancing the CRISPR/Cas9 system based on multiple GmU6 promoters in soybean. Biochem. Biophysical Res. Commun. 519 (4), 819–823. 10.1016/j.bbrc.2019.09.074 31558318

[B33] Du H. Zeng X. Zhao M. Cui X. Wang Q. Yang H. (2016). Efficient targeted mutagenesis in soybean by TALENs and CRISPR/Cas9. J. Biotechnol. 217, 90–97. 10.1016/j.jbiotec.2015.11.005 26603121

[B34] Endre G. Kereszt A. Kevei Z. Mihacea S. Kaló P. Kiss G. B. (2002). A receptor kinase gene regulating symbiotic nodule development. Nature 417 (6892), 962–966. 10.1038/nature00842 12087406

[B35] Food and Agriculture Organization (2018). *Sustainable food systems—Concept and framework. Rome: agriculture organization of the united nations* . FAO. Available online at: http://www.fao.org/3/ca2079en/CA2079EN.pdf.

[B36] Food and Agriculture Organization (2004). The state of food and agriculture 2003–2004. Rome: FAO. Available online at: https://www.fao.org/3/y5160e/Y5160E.pdf.

[B38] Gresshoff P. M. Su C. Su H. Hastwell A. Cha Y. Zhang M. (2025). Functional genomics dissection of the nodulation autoregulation pathway (AON) in soybean (Glycine max). J. Integr. Plant Biol. 67, 762–772. 10.1111/jipb.13898 40125797

[B39] Gupta S. K. Vishwakarma N. K. Malakar P. Vanspati P. Sharma N. K. Chattopadhyay D. (2023). Development of an Agrobacterium-delivered codon-optimized CRISPR/Cas9 system for chickpea genome editing. Protoplasma, 1–15. 10.1007/s00709-023-01845-2 37131068

[B40] Han J. Guo B. Guo Y. Zhang B. Wang X. Qiu L. J. (2019). Creation of early flowering germplasm of soybean by CRISPR/Cas9 technology. Front. Plant Sci. 10, 1446. 10.3389/fpls.2019.01446 31824524 PMC6882952

[B41] Haun W. Coffman A. Clasen B. M. Demorest Z. L. Lowy A. Ray E. (2014). Improved soybean oil quality by targeted mutagenesis of the fatty acid desaturase 2 gene family. Plant Biotechnol. J. 12 (7), 934–940. 10.1111/pbi.12201 24851712

[B42] Huang J. Rowe D. Subedi P. Zhang W. Suelter T. Valent B. (2022). CRISPR-Cas12a induced DNA double-strand breaks are repaired by multiple pathways with different mutation profiles in Magnaporthe oryzae. Nat. Commun. 13 (1), 7168. 10.1038/s41467-022-34736-1 36418866 PMC9684475

[B43] Indrasumunar A. Kereszt A. Searle I. Miyagi M. Li D. Nguyen C. D. (2010). Inactivation of duplicated nod factor receptor 5 (NFR5) genes in recessive loss-of-function non-nodulation mutants of allotetraploid soybean (Glycine max L. Merr.). Plant Cell Physiology 51 (2), 201–214. 10.1093/pcp/pcp178 20007291

[B44] Indrasumunar A. Searle I. Lin M. H. Kereszt A. Men A. Carroll B. J. (2011). Nodulation factor receptor kinase 1α controls nodule organ number in soybean (Glycine max L. Merr). Plant J. 65 (1), 39–50. 10.1111/j.1365-313X.2010.04398.x 21175888

[B45] Indrasumunar A. Wilde J. Hayashi S. Li D. Gresshoff P. M. (2015). Functional analysis of duplicated Symbiosis receptor kinase (*SymRK)* genes during nodulation and *mycorrhizal* infection in soybean (Glycine max). J. Plant Physiol. 176 (9), 157–168. 10.1016/j.jplph.2015.01.002 25617765

[B46] Ishii T. Araki M. (2017). A future scenario of the global regulatory landscape regarding genome-edited crops. GM crops and food 8 (1), 44–56. 10.1080/21645698.2016.1261787 27960622 PMC5592978

[B47] Jacobs T. B. LaFayette P. R. Schmitz R. J. Parrott W. A. (2015). Targeted genome modifications in soybean with CRISPR/Cas9. BMC Biotechnol. 15, 16–10. 10.1186/s12896-015-0131-2 25879861 PMC4365529

[B48] Janardhanan K. Vadivel V. Pugalenthi M. (2003). “Biodiversity in Indian under-exploited/tribal pulses,” in Improvement strategies for Leguminosae biotechnology. Editors Jaiwal, P. K. Singh R. P. (Dordrecht, Netherlands: Kluwer Academic Publishers), 353e405.

[B49] Jayachandran M. Xu B. (2019). An insight into the health benefits of fermented soy products. Food Chem. 271, 362–371. 10.1016/j.foodchem.2018.07.158 30236688

[B50] Ji J. Zhang C. Sun Z. Wang L. Duanmu D. Fan Q. (2019). Genome editing in cowpea *Vigna unguiculata* using CRISPR-Cas9. Int. J. Mol. Sci. 20 (10), 2471. 10.3390/ijms20102471 31109137 PMC6566367

[B51] Jinek M. Chylinski K. Fonfara I. Hauer M. Doudna J. A. Charpentier E. (2012). A programmable dual-RNA–guided DNA endonuclease in adaptive bacterial immunity. science 337 (6096), 816–821. 10.1126/science.1225829 22745249 PMC6286148

[B52] Joung J. K. Sander J. D. (2013). TALENs: a widely applicable technology for targeted genome editing. Nat. Rev. Mol. Cell Biol. 14, 49–55. 10.1038/nrm3486 23169466 PMC3547402

[B53] Juranić M. Nagahatenna D. S. Salinas-Gamboa R. Hand M. L. Sánchez-León N. Leong W. H. (2020). A detached leaf assay for testing transient gene expression and gene editing in cowpea (Vigna unguiculata [L.] Walp.). Plant Methods 16 (1), 88–17. 10.1186/s13007-020-00630-4 32549904 PMC7296760

[B54] Kang B. C. Yun J. Y. Kim S. T. Shin Y. Ryu J. Choi M. (2018). Precision genome engineering through adenine base editing in plants. Nat. plants 4 (7), 427–431. 10.1038/s41477-018-0178-x 29867128

[B55] Kiba T. Krapp A. (2016). Plant nitrogen acquisition under low availability: regulation of uptake and root architecture. Plant Cell Physiol. 57, 707–714. 10.1093/pcp/pcw052 27025887 PMC4836452

[B56] Kleinstiver B. P. Pattanayak V. Prew M. S. Tsai S. Q. Nguyen N. T. Zheng Z. (2016). High-fidelity CRISPR–Cas9 nucleases with no detectable genome-wide off-target effects. Nature 529 (7587), 490–495. 10.1038/nature16526 26735016 PMC4851738

[B57] Kole C. Muthamilarasan M. Henry R. Edwards D. Sharma R. Abberton M. (2015). Application of genomics-assisted breeding for generation of climate resilient crops: progress and prospects. Front. plant Sci. 6, 563. 10.3389/fpls.2015.00563 26322050 PMC4531421

[B58] Komor A. C. Kim Y. B. Packer M. S. Zuris J. A. Liu D. R. (2016). Programmable editing of a target base in genomic DNA without double-stranded DNA cleavage. Nature 533 (7603), 420–424. 10.1038/nature17946 27096365 PMC4873371

[B59] Krusell L. Madsen L. H. Sato S. Aubert G. Genua A. Szczyglowski K. (2002). Shoot control of root development and nodulation is mediated by a receptor-like kinase. Nature 420 (6914), 422–426. 10.1038/nature01207 12442170

[B60] Kuzma J. (2018). Regulating gene-edited crops. Issues Sci. Technol. 35 (1), 80–85. Available online at: https://issues.org/regulating-gene-edited-crops/.

[B61] Larweh V. Akromah R. Amoah S. Asibuo J. Y. Kusi F. Prempeh R. (2019). Effect of striga gesnerioides on cowpea (vigna unguiculata (L.) walp) yield components. Res. square 3 (5), 123–213. 10.21203/rs.3.rs-123-13/v1

[B62] Levin H. M. Pollitt E. Galloway R. McGuire J. (1993). “Micronutrient deficiency disorders,” in Disease control priorities in developing countries. Editors Jamison D. T. Mosley W. H. Measham A. R. Bobadilla J. L. 2nd ed. (Oxford (UK): Oxford University Press), 421–451.

[B63] Li C. Nguyen V. Liu J. Fu W. Chen C. Yu K. (2019). Mutagenesis of seed storage protein genes in Soybean using CRISPR/Cas9. BMC Res. notes 12, 1–7. 10.1186/s13104-019-4620-2 30917862 PMC6437971

[B64] Li C. Zong Y. Wang Y. Jin S. Zhang D. Song Q. (2018). Expanded base editing in rice and wheat using a Cas9-adenosine deaminase fusion. Genome Biol. 19, 59. 10.1186/s13059-018-1443-z 29807545 PMC5972399

[B65] Li Z. Liu Z. B. Xing A. Moon B. P. Koellhoffer J. P. Huang L. (2015). Cas9-guide RNA directed genome editing in soybean. Plant physiol. 169 (2), 960–970. 10.1104/pp.15.00783 26294043 PMC4587461

[B66] Liao W. Guo R. Qian K. Shi W. Whelan J. Shou H. (2024). The acyl–acyl carrier protein thioesterases GmFATA1 and GmFATA2 are essential for fatty acid accumulation and growth in soybean. Plant J. 118 (3), 823–838. 10.1111/tpj.16638 38224529

[B67] Liberty J. T. Poudel B. Ihedioha O. Lin H. Habanabakize E. Gao Z. (2024). Gene editing technology: shaping international standards for health and food safety assurance. Trends Biotechnol. 43, 985–988. 10.1016/j.tibtech.2024.09.019 39424445

[B68] Lin Q. Jin S. Zong Y. Yu H. Zhu Z. Liu G. (2021). High-efficiency prime editing with optimized, paired pegRNAs in plants. Nat. Biotechnol. 39 (8), 923–927. 10.1038/s41587-021-00868-w 33767395

[B69] Lin Q. Zong Y. Xue C. Wang S. Jin S. Zhu Z. (2020). Prime genome editing in rice and wheat. Nat. Biotechnol. 38, 582–585. 10.1038/s41587-020-0455-x 32393904

[B70] López C. M. Alseekh S. Martínez Rivas F. J. Fernie A. R. Prieto P. Alamillo J. M. (2025). CRISPR/Cas9 editing of two adenine phosphoribosyl transferase coding genes reveals the functional specialization of adenine salvage proteins in common bean. J. Exp. Bot., 39387692. 10.1093/jxb/erae424 PMC11714751 39387692

[B71] Lu Y. Tian Y. Shen R. Yao Q. Zhong D. Zhang X. (2021). Precise genome modification in tomato using an improved prime editing system. Plant Biotechnol. J. 19, 415–417. 10.1111/pbi.13497 33091225 PMC7955883

[B72] Luo T. Ma C. Fan Y. Qiu Z. Li M. Tian Y. (2024). CRISPR/Cas9-mediated editing of *GmARM* improves resistance to multiple stresses in soybean. Plant Sci. 346, 112147. 10.1016/j.plantsci.2024.112147 38834106

[B73] Madsen E. B. Madsen L. H. Radutoiu S. Olbryt M. Rakwalska M. Szczyglowski K. (2003). A receptor kinase gene of the LysM type is involved in legumeperception of rhizobial signals. Nature 425 (6958), 637–640. 10.1038/nature02045 14534591

[B74] Mbaya H. Lillico S. Kemp S. Simm G. Raybould A. (2022). Regulatory frameworks can facilitate or hinder the potential for genome editing to contribute to sustainable agricultural development. Front. Bioeng. Biotechnol. 10, 959236. 10.3389/fbioe.2022.959236 36246373 PMC9562833

[B75] Menz J. Modrzejewski D. Hartung F. Wilhelm R. Sprink T. (2020). Genome edited crops touch the market: a view on the global development and regulatory environment. Front. Plant Sci. 11, 586027. 10.3389/fpls.2020.586027 33163013 PMC7581933

[B76] Messina M. J. (1999). Legumes and soybeans: overview of their nutritional profiles and health effects. Am. J. Clin. Nutr. 70 (3), 439S–450s. 10.1093/ajcn/70.3.439s 10479216

[B77] Metje-Sprink Mishra (2020). Genome-edited plants in the field', Current opinion in biotechnology, 2020. 'Base editing in crops: current advances, limitations and future implications. Plant Biotechnol. J. 10.1016/j.copbio.2020.07.005 PMC6920333 31365173

[B78] Mishra R. Joshi R. K. Zhao K. (2020). Base editing in crops: current advances, limitations and future implications. Plant Biotechnol. J. 18, 20–31. 10.1111/pbi.13225 31365173 PMC6920333

[B79] Mohajan H. K. (2022). Food insecurity and malnutrition of Africa: a combined attempt can reduce them. J. Econ. Dev. Environ. People 11 (1), 24–34. 10.26458/jedep.v1i1.716

[B80] Molinari M. D. C. Pagliarini R. F. Florentino L. H. Lima R. N. Arraes F. B. M. Abbad S. V. (2024). “Navigating the path from lab to market: regulatory challenges and opportunities for genome editing technologies for agriculture,” in Plant genome editing technologies, 25–63. 10.1007/978-3-031-52361-4_2

[B81] Nabhan G. P. (2016). Enduring seeds: native American agriculture and wild plant conservation. Tucson, AZ: University of Arizona Press.

[B82] Nishida K. Arazoe T. Yachie N. Banno S. Kakimoto M. Tabata M. (2016). Targeted nucleotide editing using hybrid prokaryotic and vertebrate adaptive immune systems. Science 353 (6305), aaf8729. 10.1126/science.aaf8729 27492474

[B83] Nishimura R. Hayashi M. Wu G. J. Kouchi H. Imaizumi-Anraku H. Murakami Y. (2002). HAR1 mediates systemic regulation of symbiotic organ development. Nature 420 (6914), 426–429. 10.1038/nature01231 12442172

[B84] Nonaka S. Arai C. Takayama M. Matsukura C. Ezura H. (2017). Efficient increase of ɣ-aminobutyric acid (GABA) content in tomato fruits by targeted mutagenesis. Sci. Rep. 7 (1), 7057. 10.1038/s41598-017-06400-y 28765632 PMC5539196

[B85] Parisi C. Rodríguez-Cerezo E. (2021). Current and future market applications of new genomic techniques. Luxembourg: Publications Office of the European Union.JRC123830

[B86] Peng R. Lin G. Li J. (2016). Potential pitfalls of CRISPR/Cas9‐mediated genome editing. FEBS J. 283 (7), 1218–1231. 10.1111/febs.13586 26535798

[B87] Peoples M. B. Giller K. E. Jensen E. S. Herridge D. F. (2021). Quantifying country-to-global scale nitrogen fixation for grain legumes: I. Reliance on nitrogen fixation of soybean, groundnut and pulses. Plant Soil 469, 1–14. 10.1007/s11104-021-05167-6

[B88] Peters D. (2021). Public awareness and understanding of gene edited foods in the US. Ames, Iowa: Iowa State University. Available online at: https://store.extension.iastate.edu/product/Public-Awareness-and-Understanding-of-Gene-Edited-Foods-in-the-US (Accessed April 30, 2021).

[B89] Petersen B. L. Möller S. R. Mravec J. Jørgensen B. Christensen M. Liu Y. (2019). Improved CRISPR/Cas9 gene editing by fluorescence activated cell sorting of green fluorescence protein tagged protoplasts. BMC Biotechnol. 19, 36. 10.1186/s12896-019-0530-x 31208390 PMC6580576

[B90] Piatek A. Ali Z. Baazim H. Li L. Abulfaraj A. Al‐Shareef S. (2015). RNA‐guided transcriptional regulation in planta via synthetic dC as9‐based transcription factors. Plant Biotechnol. J. 13 (4), 578–589. 10.1111/pbi.12284 25400128

[B91] Pinstrup–Andersen P. Burger S. Habicht J. P. Peterson K. (1993). “Protein–energy malnutrition,” in Disease control priorities in developing countries. Editors Jamison D. T. Mosley W. H. Measham A. R. Bobadilla J. L. 2nd ed. (Oxford (UK): Oxford University Press), 391–420.

[B92] Qin L. Li J. Wang Q. Xu Z. Sun L. Alariqi M. (2020). High-efficient and precise base editing of C⋅G to T⋅A in the allotetraploid cotton (Gossypium hirsutum) genome using a modified CRISPR/Cas9 system. Plant Biotechnol. J. 18, 45–56. 10.1111/pbi.13168 31116473 PMC6920158

[B93] Rose K. M. Brossard D. Scheufele D. A. (2020). Of society, nature, and health: how perceptions of specific risks and benefits of genetically engineered foods shape public rejection. Environ. Commun. 14 (7), 1017–1031. 10.1080/17524032.2019.1710227

[B94] Roy A. Sandhu R. (2024). Advancements in genetic enhancement: CRISPR/Cas-Mediated genome editing in leguminous crops. J. Adv. Biol. and Biotechnol. 27 (6), 670–681. 10.1000/jabb.2024.0670

[B95] Ryan E. Galvin K. O’Connor T. P. Maguire A. R. O’Brien N. M. (2007). Phytosterol, squalene, tocopherol content and fatty acid profile of selected seeds, grains, and legumes. Plant Foods Hum. Nutr. 62, 85–91. 10.1007/s11130-007-0046-8 17594521

[B96] Salgado J. M. Donado-Pestana C. M. (2011). “Soy as a functional food,” in Soybean and nutrition, 21–44.

[B97] Samaranayaka A. (2017). “Lentil: revival of poor man’s meat,” in Sustainable protein sources (Academic Press), 185–196.

[B98] Sarker R. H. Das S. K. Shethi K. J. Hoque M. I. (2019). “Genetic transformation,” in Lentils (Academic Press), 141–202.

[B99] Sarker R. H. Mustafa B. M. Biswas A. Mahbub S. Hoque M. I. (2003). Agrobacterium-mediated transformation of lentil (Lens culinaris Medik.). Plant Tissue Cult. 13 (1), 1–12. 10.3329/ptcb.v22i1.11243

[B100] Sauer Mozoruk J. Miller R. B. Warburg Z. J. Walker K. A. Beetham P. R. (2016). Oligonucleotide‐directed mutagenesis for precision gene editing. Plant Biotech. J. 14, 496–502. 10.1111/pbi.12496 PMC5057361 26503400

[B101] Scherer S. Davis R. W. (1979). Replacement of chromosome segments with altered DNA sequences constructed *in vitro* . Proc. Natl. Acad. Sci. 76 (10), 4951–4955. 10.1073/pnas.76.10.4951 388424 PMC413056

[B102] Schnabel E. Journet E. P. de Carvalho-Niebel F. Duc G. Frugoli J. (2005). The Medicago truncatula SUNN gene encodes a CLV1-like leucine-rich repeat receptor kinase that regulates nodule number and root length. Plant Mol. Biol. 58 (6), 809–822. 10.1007/s11103-005-8102-y 16240175

[B103] Schofield G. Henderson G. (2015). Red meat, processed meat, and cancer–how strong is the evidence.

[B104] Searle I. R. Men A. E. Laniya T. S. Buzas D. M. Iturbe-Ormaetxe I. Carroll B. J. (2003). Long-distance signaling in nodulation directed by a CLAVATA1-like receptor kinase. Science 299 (5603), 109–112. 10.1126/science.1077937 12411574

[B105] Shimatani Z. Kashojiya S. Takayama M. Terada R. Arazoe T. Ishii H. (2017). Targeted base editing in rice and tomato using a CRISPR-Cas9 cytidine deaminase fusion. Nat. Biotechnol. 35, 441–443. 10.1038/nbt.3833 28346401

[B106] Shu H. Luo Z. Peng Z. Wang J. (2020). The application of CRISPR/Cas9 in hairy roots to explore the functions of AhNFR1 and AhNFR5 genes during peanut nodulation. BMC plant Biol. 20 (1), 1–15. 10.1186/s12870-020-02455-y 32894045 PMC7487912

[B107] Siddhuraju P. Becker K. (2003). Studies on antioxidant activities of Mucuna seed (Mucuna pruriens var. utilis) extract and various non-protein amino/imino acids through *in vitro* models. J. Sci. Food Agric. 83, 1517–1524. 10.1002/jsfa.1587

[B108] Singer S. D. Mukthar M. M. Subedi U. Poudel H. Chen G. Foroud N. (2024). CRISPR/Cas-mediated gene editing in plant immunity and its potential for the future development of fungal, oomycete, and bacterial pathogen-resistant pulse crops. Plant, Cell and Environ. 10.1111/pce.15174 39351611

[B109] Singh J. P. Singh B. Kaur A. (2021). “Bioactive compounds of legume seeds,” in Bioactive compounds in underutilized vegetables and legumes, 645–665.

[B110] Slaymaker I. M. Gao L. Zetsche B. Scott D. A. Yan W. X. Zhang F. (2016). Rationally engineered Cas9 nucleases with improved specificity. Science 351 (6268), 84–88. 10.1126/science.aad5227 26628643 PMC4714946

[B111] Smit P. Limpens E. Geurts R. Fedorova E. Dolgikh E. Gough C. (2007). Medicago LYK3, an entry receptor in rhizobial nodulation factor signaling. Plant physiol. 145 (1), 183–191. 10.1104/pp.107.100495 17586690 PMC1976573

[B112] Smyth S. J. (2020). Regulatory barriers to improving global food security. Glob. food Secur. 26, 100440. 10.1016/j.gfs.2020.100440 PMC7521901 33014703

[B113] Song B. Luo T. Fan Y. Li M. Qiu Z. Tian Y. (2024). Generation of new β-conglycinin-deficient soybean lines by editing the lincRNA lincCG1 using the CRISPR/Cas9 system. J. Agric. Food Chem. 72 (26), 15013–15026. 10.1021/acs.jafc.4c02269 38907729

[B114] Songstad D. D. Petolino J. F. Voytas D. F. Reichert N. A. (2017). Genome editing of plants. Crit. Rev. Plant Sci. 36 (1), 1–23. 10.1080/07352689.2017.1359564

[B115] Stagnari F. Maggio A. Galieni A. Pisante M. (2017). Multiple benefits of legumes for agriculture sustainability: an overview. Chem. Biol. Technol. Agric. 4, 2–13. 10.1186/s40538-016-0085-1

[B116] Stracke S. Kistner C. Yoshida S. Mulder L. Sato S. Kaneko T. (2002). A plant receptor-like kinase required for both bacterial and fungal symbiosis. Nature 417 (6892), 959–962. 10.1038/nature00841 12087405

[B117] Stupar R. M. Specht J. E. (2013). Insights from the soybean (Glycine max and Glycine soja) genome: past, present, and future. Adv. Agron. 118, 177–204. 10.1016/B978-0-12-405942-9.00005-9

[B118] Sulieman S. Tran L. S. P. (2015). “Introduction,” in Legume nitrogen fixation in a changing environment: achievements and challenges. Editors Sulieman, S. Tran L. S. P. (Switzerland: Springer International Publishing), 1–3. 10.1007/978-3-319-06212-0_1

[B119] Sun X. Hu Z. Chen R. Jiang Q. Song G. Zhang H. (2015). Targeted mutagenesis in soybean using the CRISPR-Cas9 system. Sci. Rep. 5 (1), 10342. 10.1038/srep10342 26022141 PMC4448504

[B120] Tang X. Sretenovic S. Ren Q. Jia X. Li M. Fan T. (2020). Plant prime editors enable precise gene editing in rice cells. Mol. plant 13 (5), 667–670. 10.1016/j.molp.2020.03.010 32222487

[B121] Vadivel V. Pugalenthi M. (2008). Effect of various processing methods on the antinutritional constituents and protein digestibility of velvet bean seeds. J. Food Biochem. 32, 795e812. 10.1111/j.1745-4514.2008.00199.x

[B122] Vasconcelos M. W. Gomes A. M. (2016). The legume grains: when tradition goes hand in hand with nutrition. Traditional Foods General Consumer Aspects, 189–208. 10.1007/978-1-4899-7648-2_13

[B123] Veillet F. Kermarrec M. P. Chauvin L. Guyon-Debast A. Chauvin J. E. Gallois J. L. (2020). Prime editing is achievable in the tetraploid potato, but needs improvement. BioRxiv, 2020–2106. 10.1101/2020.08.04.237040

[B124] Verma A. Kaur L. Kaur N. Bhardwaj A. Pandey A. K. Kandoth P. K. (2024). Genome editing of an oxalyl-CoA synthetase gene in *Lathyrus sativus* reveals its role in oxalate metabolism. Plant Cell Rep. 43 (12), 280. 10.1007/s00299-024-03368-8 39538000

[B125] Virdi K. S. Spencer M. Stec A. O. Xiong Y. Merry R. Muehlbauer G. J. (2020). Similar seed composition phenotypes are observed from CRISPR-generated in-frame and knockout alleles of a soybean KASI ortholog. Front. Plant Sci. 11, 1005. 10.3389/fpls.2020.01005 32774339 PMC7381328

[B126] Wang J. Kuang H. Zhang Z. Yang Y. Yan L. Zhang M. Guan Y. (2020). Generation of seed lipoxygenase-free soybean using CRISPR-Cas9. Crop J. 8 (3), 432–439. 10.1016/j.cj.2020.01.007

[B127] Warkentin T. D. McHughen A. (1993). Regeneration from lentil cotyledonary nodes and potential of this explant for transformation by *Agrobacterium tumefaciens* . Lens Newsl. 20 (1), 26–28. 10.1017/CBO9781316018874

[B128] Wells J. C. (2016). *The metabolic ghetto: An evolutionary perspective on nutrition, power relations and chronic disease [Front matter*]. Cambridge University Press. Available online at: https://assets.cambridge.org/97811070/09479/frontmatter/9781107009479_frontmatter.pdf.

[B129] Willett W. Rockström J. Loken B. Springmann M. Lang T. Vermeulen S. (2019). Food in the Anthropocene: the EAT–Lancet Commission on healthy diets from sustainable food systems. lancet 393 (10170), 447–492. 10.1016/S0140-6736(18)31788-4 30660336

[B130] Wood J. A. Grusak M. A. (2007). “Nutritional value of chickpea,” in Chickpea breeding and management (Wallingford UK: CABI), 101–142.

[B131] World Health Organization (2004). Vitamin and mineral requirements in human nutrition. 2nd ed. Geneva: WHO. Available online at: https://www.who.int/publications/i/item/9241546123.

[B132] Wu N. Lu Q. Wang P. Zhang Q. Zhang J. Qu J. (2020). Construction and analysis of GmFAD2-1A and GmFAD2-2A soybean fatty acid desaturase mutants based on CRISPR/Cas9 technology. Int. J. Mol. Sci. 21 (3), 1104. 10.3390/ijms21031104 32046096 PMC7037799

[B133] Xiang X. Yang H. Yuan X. Dong X. Mai S. Zhang Q. (2024). CRISPR/Cas9-mediated editing of *GmDWF1* brassinosteroid biosynthetic gene induces dwarfism in soybean. Plant Cell Rep. 43, 116. 10.1007/s00299-024-03204-z 38622229

[B134] Yao Z. D. Cao Y. N. Peng L. X. Yan Z. Y. Zhao G. (2020). Coarse cereals and legume grains exert beneficial effects through their interaction with gut microbiota: a review. J. Agric. Food Chem. 69 (3), 861–877. 10.1021/acs.jafc.0c05691 33264009

[B135] Yitbarek M. B. (2019). Livestock and livestock product trends by 2050. IJAR 4, 30. Available online at: https://escipub.com/pdf/IJAR-2019-07-2305.pdf

[B136] Yuan M. Zhu J. Gong L. He L. Lee C. Han S. He G. (2019). Mutagenesis of FAD2 genes in peanut with CRISPR/Cas9 based gene editing. BMC Biotechnol. 19 (1), 1–7. 31035982 10.1186/s12896-019-0516-8 PMC6489235

[B137] Zaheer K. Humayoun Akhtar M. (2017). An updated review of dietary isoflavones: nutrition, processing, bioavailability and impacts on human health. Crit. Rev. food Sci. Nutr. 57 (6), 1280–1293. 10.1080/10408398.2014.989958 26565435

[B138] Zhang X. Ferreira I. R. S. Schnorrer F. (2014). A simple TALEN-based protocol for efficient genome-editing in Drosophila. Methods 69 (1), 32–37. 10.1016/j.ymeth.2014.03.020 24680697

[B139] Zhao Y. Cheng P. Liu Y. Liu C. Hu Z. Xin D. (2025). A highly efficient soybean transformation system using GRF3-GIF1 chimeric protein. J. Integr. Plant Biol. 67 (1), 3–6. 10.1111/jipb.13767 39240004 PMC11734085

[B140] Zheng N. Li T. Dittman J. D. Su J. Li R. Gassmann W. (2020). CRISPR/Cas9-based gene editing using egg cell-specific promoters in Arabidopsis and soybean. Front. Plant Sci. 11, 800. 10.3389/fpls.2020.00800 32612620 PMC7309964

[B141] Zhong X. Wang J. Shi X. Bai M. Yuan C. Cai C. (2024). Genetically optimizing soybean nodulation improves yield and protein content. Nat. Plants 10 (5), 736–742. 10.1038/s41477-024-01696-x 38724696

[B142] Zhong Z. Liu C. Liu B. Wang X. Li Y. Xie S. (2022). Plant genome editing using prime editing: progress and challenges. Plant Commun. 3 (2), 100252. 10.1016/j.xplc.2022.100252

[B37] Zong Y. Wang Y. Li C. Zhang R. Chen K. Ran Y. (2017). Precise base editing in rice, wheat and maize with a Cas9-cytidine deaminase fusion. Nat. Biotechnol. 35, 438–440. 10.1038/nbt.3811 28244994

