# Supplementary material for: Genome editing in grain legumes for food security
Source: Front Genome Ed. 2025 May 20;7:1572292. doi: 10.3389/fgeed.2025.1572292 (PMC12140438; doi:10.3389/fgeed.2025.1572292)
Supplement: Supplementary file 1 [file Supplementaryfile1.docx]

**Supplementary data**

*Search strings in web of Science and Scopus:* ("CRISPR/Cas9" OR "CRISPR Cas9" OR "CRISPR" OR "CRISPR/Cas9-mediated genome editing") AND ("grain legumes" OR "soybeans" OR "cowpea" OR "chickpea" OR "peanut" OR "leguminous crops")


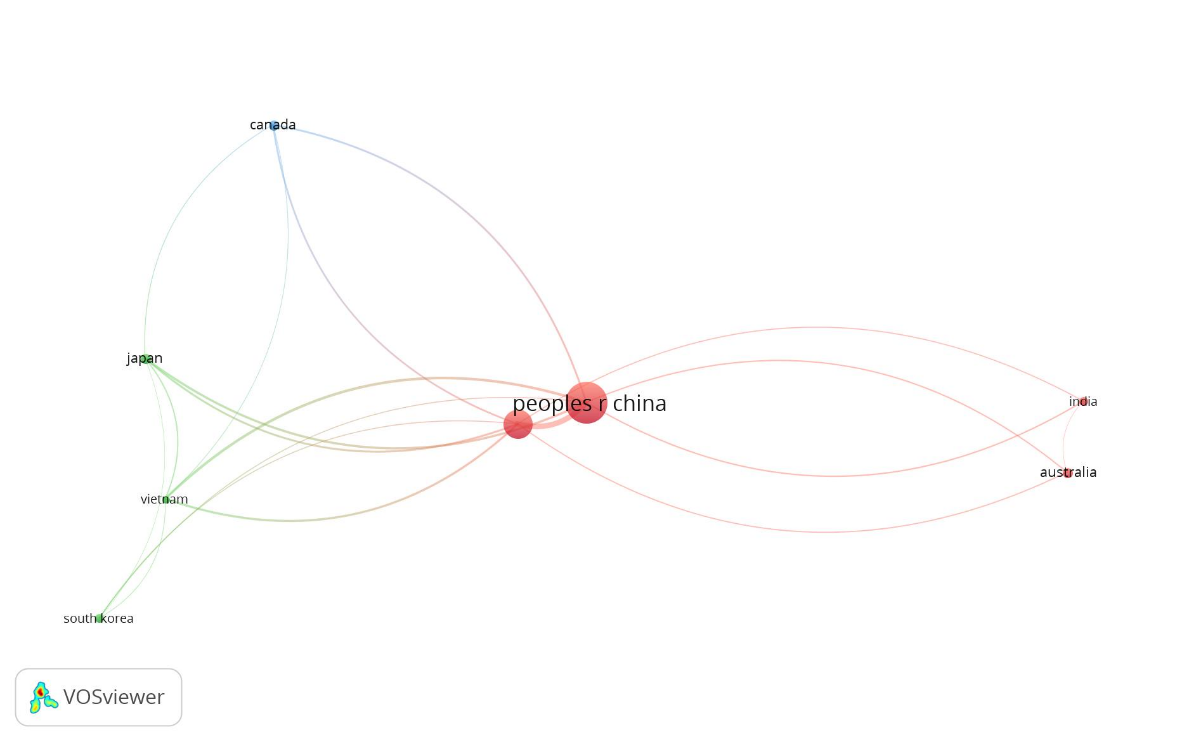


**Figure 1: Country Level Bibliographic coupling**


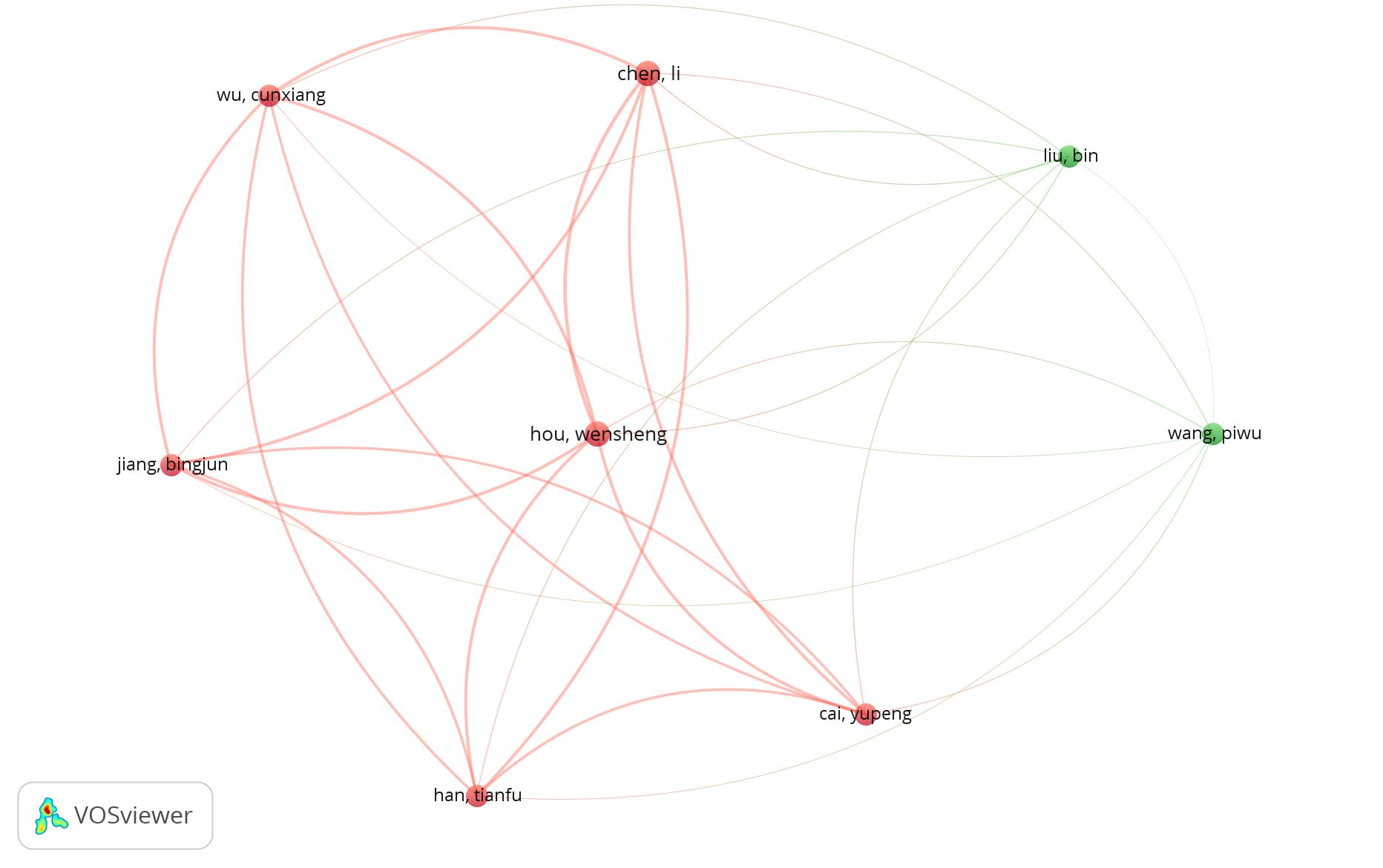


**Figure 2: Researcher-level Bibliographic coupling**
